# LPEATs Tailor Plant Phospholipid Composition through Adjusting Substrate Preferences to Temperature

**DOI:** 10.3390/ijms22158137

**Published:** 2021-07-29

**Authors:** Sylwia Klińska, Kamil Demski, Katarzyna Jasieniecka-Gazarkiewicz, Antoni Banaś

**Affiliations:** Intercollegiate Faculty of Biotechnology, University of Gdansk and Medical University of Gdansk, 80-307 Gdansk, Poland; katarzyna.jasieniecka@biotech.ug.edu.pl (K.J.-G.); antoni.banas@biotech.ug.edu.pl (A.B.)

**Keywords:** LPEAT, LPLAT, phospholipid, abiotic stress, heat stress, cold stress, *Camelina sativa*

## Abstract

Acyl-CoA:lysophosphatidylethanolamine acyltransferases (LPEATs) are known as enzymes utilizing acyl-CoAs and lysophospholipids to produce phosphatidylethanolamine. Recently, it has been discovered that they are also involved in the growth regulation of *Arabidopsis thaliana*. In our study we investigated expression of each *Camelina sativa* LPEAT isoform and their behavior in response to temperature changes. In order to conduct a more extensive biochemical evaluation we focused both on LPEAT enzymes present in microsomal fractions from *C. sativa* plant tissues, and on cloned *Cs*LPEAT isoforms expressed in yeast system. Phylogenetic analyses revealed that *Cs*LPEAT1c and *Cs*LPEAT2c originated from *Camelina hispida*, whereas other isoforms originated from *Camelina neglecta*. The expression ratio of all CsLPEAT1 isoforms to all CsLPEAT2 isoforms was higher in seeds than in other tissues. The isoforms also displayed divergent substrate specificities in utilization of LPE; CsLPEAT1 preferred 18:1-LPE, whereas *Cs*LPEAT2 preferred 18:2-LPE. Unlike *Cs*LPEAT1, *Cs*LPEAT2 isoforms were specific towards very-long-chain fatty acids. Above all, we discovered that temperature strongly regulates LPEATs activity and substrate specificity towards different acyl donors, making LPEATs sort of a sensor of external thermal changes. We observed the presented findings not only for LPEAT activity in plant-derived microsomal fractions, but also for yeast-expressed individual *Cs*LPEAT isoforms.

## 1. Introduction

Terrestrial plants are continually subjected to diverse environmental stimuli. One of the biggest challenges facing worldwide flora is exposure to a broad range of temperatures. Current ambient temperature conditions are characterized by frequent fluctuations and constant increase due to climate change. High and low temperatures may cause diverse detrimental effects on plant physiology, e.g., the inhibition of protein folding or its enzymatic activity, increase in oxidative stress and ROS generation, and impairment of photosynthesis. This may ultimately lead to reduced plant growth, yield, or even cell death [1]. Both heat and cold also influence cell membrane fluidity. Proper membrane structure is required to maintain homeostasis and protect the membrane itself and its proteins from deleterious temperature effects [2,3,4]). The upkeep of membrane fluidity appropriate to surrounding environmental conditions is mainly regulated by phospholipids and their structure: either a liquid crystalline or a solid phase (also known as gel phase). Liquid structure is promoted by low temperature and is composed by unsaturated fatty acids, whereas solid phase is promoted by heat stress and favors saturated and long-chain fatty acids [5]. One of the major phospholipids composing cell membranes is phosphatidylethanolamine (PE).

PE is one of the main eukaryotic membrane-building components, constituting—together with phosphatidylcholine (PC) approximately 50% of all cellular phospholipids [6,7]. Much research has provided significant information regarding PE’s physiological role in prokaryotic and eukaryotic cells. PE may be crucial in spatial structure formation, by creating hexagonal structures [8], maintaining the proper activity of yeast respiratory system and controlling the correct structure, folding, and activity of peripheral membrane proteins [9]. PE-derivatives also play a pivotal role as second messengers [10]. Additionally, Rockenfeller et al. [11] reported that high levels of PE may positively regulate autophagy process and prolong longevity in yeast and flies. Other studies established the essential role of PE in cell death via DHA-dependent pathway and via ferroptosis [12,13].

In eukaryotic cells, three different pathways of PE synthesis can be distinguished. The compound is synthesized mainly *via* the CDP-ethanolamine pathway including three successive reactions described by Kennedy and Weiss [14]. First, PE is phosphorylated by ethanolamine kinase (EK) in ATP-dependent reaction. Subsequently, PE is conjugated with CTP to form a high-energy donor—CDP-ethanolamine. This step is catalyzed by CTP:phosphoethanolamine cytidyltransferase (ECT). The last reaction is conducted by CDP-ethanolamine:1,2-diacylglycerol ethanolaminephosphotransferase (EPT) responsible for transferring ethanolamine from CDP-ethanolamine to *sn-*3 position of diacylglycerol, providing a final product—phosphatidylethanolamine. The second possible way of PE biosynthesis is *via* the phosphatidylserine decarboxylase (PSD) activity, where serine is converted into ethanolamine by detachment of a carboxyl group [15]. The biosynthesis of PE in plant cells may also occur via acyl-CoA: lysophosphatydylethanolamine acyltransferases activity (LPEAT; [16]).

The biochemical role of LPEAT (a representative of acyl-CoA:lysophospholipid acyltransferases—LPLATs) is to transfer acyl group from a cytosolic pool of acyl-CoA to lysophosphatydylethanolamine, producing PE [17]. LPLAT enzymes can also conduct reverse reactions supplying acyl pool with newly modified/synthesized fatty acids, therefore playing an essential role in acyl editing. PC-synthesizing LPCAT (acyl-CoA:lysofosfatydylcholine acyltransferases) enzymes are recognized as major contributors to the acyl editing process (and subsequently to the storage lipid composition) both through their forward and reverse actions [18] (Bates et al. 2012). The substrate specificity and efficiency of both LPLAT reactions may significantly vary [19,20]. In case of LPEAT enzymes present in *Camelina sativa* seeds, their contribution to the remodeling of acyl-CoA pool is rather small compared to the activity of LPCAT (acyl-CoA:lysofosfatydylcholine acyltransferases) enzymes, which seems to play a major role in this process. Deacylation activity of LPLAT enzymes may also be effectively supported by phospholipase A_2_ in a process called Land’s cycle [21] as well as with PDAT (phospholipid:diacylglycerol acyltransferases) type of enzymes [22].

The knowledge about plant LPLAT enzymes is still insufficient to fully understand their biochemical and physiological role. Their main function is the regulation of the composition of phospholipid molecular species present in cell membranes. Therefore, they are responsible for maintaining proper membrane fluidity, structure, and vesicle trafficking [23]. Until now, most studies have focused on LPCAT or LPAAT (acyl-CoA:lysophosphatidic acid acyltransferases) enzymes, which synthesize phosphatidylcholine and phosphatidic acid, respectively.

LPEAT enzymes were first characterized in *Arabidopsis thaliana*. In 2009, two genes, At1g80950 and At2g45670, were recognized as genes encoding proteins with LPEAT activity: LPEAT1 and LPEAT2 isoenzymes [16]. Later studies concerning their physiological role revealed that both genes are necessary for proper growth and development. The T-DNA insertion mutant *lpeat2* and double mutant (*lpeat1 lpeat2*) of *A. thaliana* were characterized by impaired growth, shorter roots, smaller leaves, and reduced lipid content; especially, the content of PE was lowered. The opposite morphological results were observed for overexpressors [24]. Furthermore, recent study demonstrated that inhibiting LPEAT activity may prolong plant lifespan and delay the senescence process [25].

Despite the much smaller contribution of LPEAT compared to LPCAT in acyl-CoA pool editing, these enzymes may hold key roles in other significant physiological processes. In the presented research, we propose a new physiological role of LPEAT enzymes: responding to temperature variations with modifications of the membrane phospholipid make-up. In *in vitro* assays, we found changes in the substrate specificity of LPEATs (present in microsomal fractions derived from different vegetative and generative tissues) subjected to various temperature conditions. Additionally, we established the expression of each homeolog encoding LPEAT1 and LPEAT2 isoenzymes. Furthermore, we undertook the challenge of cloning genes encoding LPEAT enzymes’ homeologs from a hexaploid plant (*Camelina sativa*). Isolated genes encoding appropriate variants of LPEAT isoenzymes were biochemically characterized to determine both their specificity towards different fatty acid donors (acyl-CoAs) and acceptors (lysolipids), and how their properties are influenced by thermal conditions.

## 2. Results

### 2.1. Temperature Alters Acyl-CoA Specificity of LPEAT Enzymes Present in Vegetative Tissues

To establish the influence of temperature on acyl-CoA specificities of LPEAT enzymes, we used microsomal fractions isolated from different tissues: one generative (seeds) and three vegetative (leaves cultivated in vitro, leaves cultivated in vivo, and roots cultivated in vitro). The studied temperature ranged between 10 °C and 40 °C. Prior to major analysis, optimization in the search of the best parameters for enzymatic in vitro reaction was carried out. In case of seeds optimal reaction parameters were determined in previous study done by Klińska et al. [26]. For vegetative tissues, this optimization was conducted on microsomal fractions derived from in vivo leaves. Based on both evaluations the chosen in vitro reaction parameters were microsomal fraction equivalent to 0.5 nmol of endogenous PC per assay, a reaction time of 60 min and buffer pH equal to 7.2. The chosen pH corresponds to pH in plant endoplasmic reticulum [27] and was chosen despite the fact that for in vivo leaves optimal pH value was 9.0 and for seeds the optimal pH value stretched between 9.0 and 10.0 (Supplementary Figure S1; [28]. We decided to evaluate LPEAT acyl-CoA specificity with 5 substrates: 16:0-CoA, 18:0-CoA, 18:1-CoA, 18:2-CoA, and 18:3-CoA. Acyl-CoAs we picked constitute the most common group of fatty acids found in PE pools of various plant tissues.

**Figure 1:**
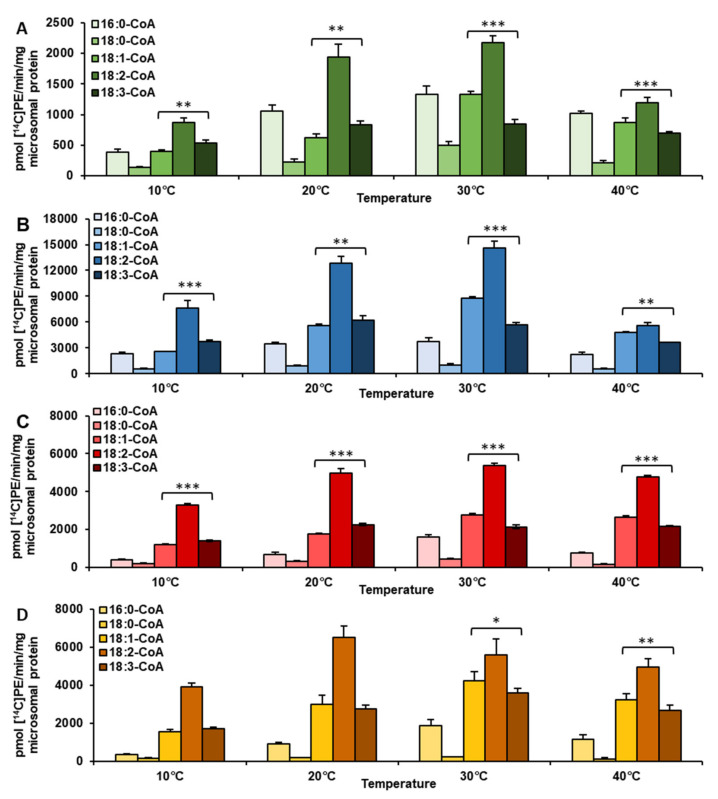
Activity of acyl-CoA:lysophospatidylethanolamine acyltransferases (LPEATs) present in microsomal fractions of *Camelina sativa* tissues towards five acyl-CoAs at different temperatures. ((**A**)—in vivo leaves; (**B**)—in vitro leaves; (**C**)—n vitro roots; (**D**)—seeds). Mean values and SD are presented (data from at least three independent assays). Asterisks denote significant differences between activity towards 18:1-CoA and 18:3-CoA at different temperatures in a mean difference two-sided Student’s t test: * *p* ≤ 0.05; ** *p* ≤ 0.01; *** *p* ≤ 0.001.

Taking into account all accumulated results presented in Figure 1, we noticed that activity towards 18:2-CoA was the highest regardless of the tissue analyzed. The only divergence occurred in regard to temperature. LPEAT reached the highest activity (towards 18:2-CoA) at 30 °C for vegetative tissue and at 20 °C for seeds. The enzyme showed the least specificity towards 18:0-CoA. The preference towards the 18:0 acyl group reached at most 11% (except microsomal fractions from in vivo leaves where this value rose to about 23%) of the LPEAT activity towards 18:2-CoA. Temperature change did not affect the enzyme’s overall activity drastically. The top values, however, were obtained at 30 °C for each analyzed tissue.

LPEAT enzymes present in in vivo leaves exhibited elevated preference (compared to other analyzed tissues) not only towards 18:0-CoA (see above) but also towards 16:0-CoA. At 20, 30, and 40 °C it was the second most favored acyl donor. LPEAT activity with 16:0-CoA reached respectively: 54.4%, 60% and 84.7% of its activity towards 18:2-CoA, at the aforementioned temperatures (Figure 1A).

In other tested tissues, LPEAT preferred 18:1-CoA and 18:3-CoA over 16:0-CoA. Which of the two acyl-CoAs LPEAT was more specific towards depended on temperature. This was especially true in vegetative tissues. At 10 and 20 °C LPEAT enzymes from vegetative tissues preferentially utilized 18:3-CoA with the efficiency ranging between 111 and 146% of LPEAT activity towards 18:1-CoA. The results were opposite at higher tested temperatures, where 18:1-CoA was more favored by LPEAT enzymes. LPEAT activity with 18:1-CoA reached about 120–130% of LPEAT activity with 18:3-CoA at 40 °C. At 30 °C the differences were even bigger and oscillated between 130 and 158% of LPEAT activity towards 18:3-CoA (Figure 1A–C). Such fluctuations in preference towards oleic and linolenic acids were also noticed in seeds, however only at 30 and 40 °C. At these temperatures LPEAT activity with 18:3-CoA achieved only between 77 and 85% of its activity towards 18:1-CoA (Figure 1D).

Not only LPEAT, but also LPCAT enzymes are able to synthesize phosphatidylethanolamine to some extent [20]. To exclude the possibility that the observed effects were a result of LPCAT enzymes activity, we conducted LPCAT substrate specificity assays towards various acyl donors at different temperatures in two different tissues (in vivo leaves and in vitro roots). The LPEAT-like trend of substrate specificity changing with reaction temperature could not be observed for LPCAT enzyme assays. Both analyzed enzymes exhibited a distinct pattern of substrate specificity towards tested acyl donors (Figure 1; Supplementary Figure S2).

The lowest activity of LPEAT enzymes, for all tested acyl donors, was detected in microsomal fractions prepared from in vivo leaves. The highest LPEAT activity was observed for in vitro leaves. For example, LPEAT activity towards 18:2-CoA in in vitro leaves was 6.6 times higher in comparison to in vivo leaves at 20 and 30 °C. At 10 °C the difference between the tissues was even more pronounced—8.8 times higher. However, at 40 °C, LPEAT activity in in vitro leaves was only 4.7 times higher than in leaves in vivo (Figure 1A,B).

**Figure 2:**
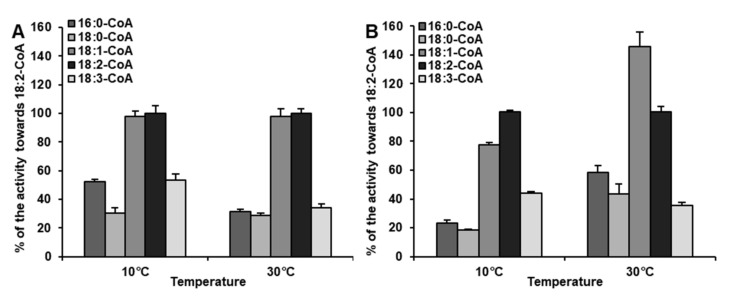
Activity of acyl-CoA:lysophospatidylethanolamine acyltransferases (LPEATs) present in microsomal fraction of *Camelina sativa* derived from in vivo (**A**) and in vitro (**B**) leaves towards five various acyl-CoAs added in equimolar concentrations with BSA into the reaction mixture. Mean values and SD are presented (data from at least three independent assays).

Microsomal fractions from both kinds of leave tissues were used in substrate selectivity assays to verify the results observed in single acyl-CoA assays, described above. The assays were conducted at 10 and 30 °C—temperatures at which significant changes in substrate specificity of LPEAT were observed. BSA was added to enzyme assays to replace the naturally occurring acyl-CoA binding protein (ACBP). In these assays LPEAT enzymes from microsomal fractions of in vivo leaves, showed almost equal preference towards 18:1-CoA and 18:2-CoA, regardless of temperature. The activity towards 16:0-CoA, 18:0-CoA and 18:3-CoA at 30 °C was almost equal and accounted for about 30% of that towards 18:2-CoA. At 10 °C relative affinity towards 18:0-CoA remained similar, whereas towards 16:0-CoA and 18:3-CoA increased to around 50% of the relative affinity towards 18:2-CoA (Figure 2B). The changes in acyl-CoA preferences of LPEAT enzymes from microsomal fractions of in vitro leaves were less pronounced than those described above. However, also in these assays, the preferences of LPEAT enzymes towards 18:1-CoA were more elevated compared to single acyl-CoA assays especially at 30 °C. At that temperature the LPEATs activity towards 18:1-CoA reached about 145% of that towards 18:2-CoA. The relative activity towards 18:3-CoA compared to 18:2-CoA was somewhat similar like in single acyl-CoA assays with somewhat higher affinity to 18:3-CoA at 10 °C compared to 30 °C. Unlike in single acyl-CoA assays the LPEAT affinity towards 16:0-CoA and 18:0-CoA was similar and at 10 °C accounted for about 20% of the LPEAT affinity towards 18:2-CoA, whereas at 30 °C their activity raised to 59% and 43%, respectively (Figure 1A; Figure 2A).

Additionally, we conducted assays determining the rate of incorporation of fatty acids from acyl-CoA pool to PE of microsomal fractions of in vivo leaves. Using these results, we also estimated the time of complete fatty acid turnover in PE. The assays were conducted in two extreme temperatures: 10 and 40 °C. In such assays, fatty acids from acyl-CoA pool were incorporated into lyso-PE (LPE) via LPEAT action and PE with new set of fatty acids was created. In the assay conditions we used, the LPE available for LPEAT is formed via phospholipase A activity, via PDAT activiaty and via the reverse reaction of LPEAT enzymes. Thus, the results constitute the rate of fatty acids remodeling of PE with participation of all of the above mentioned types of reactions. The observed intensity of remodeling of fatty acids of PE was from 2.7 to 4.9 times faster at 40 °C than at 10 °C. The biggest difference was observed, if 18:2-CoA was used in the assays and the lowest difference was observed, if 18:3-CoA was used. At 10 °C, the estimated complete turnover of PE fatty acids took about 7, 9, and 14 days and at 40 °C about 2, 2, and 5 days for 18:1-CoA, 18:2-CoA and 18:3-CoA fatty acid donors, respectively (Table 1).

**Figure 3:**
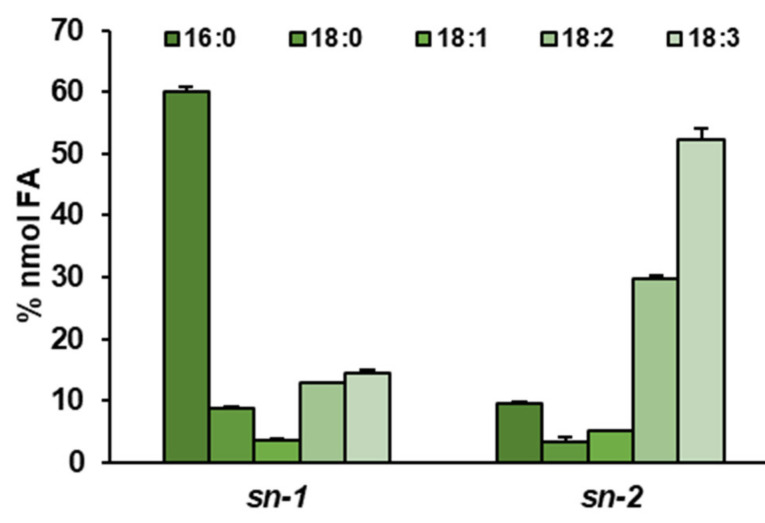
Fatty acids composition of *sn-1* and *sn-2* position in phosphatidylethanolamine isolated from *C. sativa* in vivo leaves. Error bars indicate the SD between the three biological repeats (*n* = 3).

Next, we determined the fatty acid composition of sn-1 and sn-2 positions in PE of *Camelina sativa* in vivo leaves. At the sn-1 palmitic acid (16:0) was the most dominant fatty acid, followed by linolenic (18:3) and linoleic (18:2) acids, accounting for respectively 60, 15, and 13% of all fatty acids present at sn-1 position. The relative amounts of 18:0 and 18:1 accounted for about 8 and 4%, respectively. The most abundant acyl groups at sn-2 position were polyunsaturated fatty acids 18:3 and 18:2 followed by 16:0—53, 30, and 10%, respectively (Figure 3).

### 2.2. Evolutionary Analyses Reveal Ancestral Origins of Camelina sativa LPEATs

**Figure 4:**
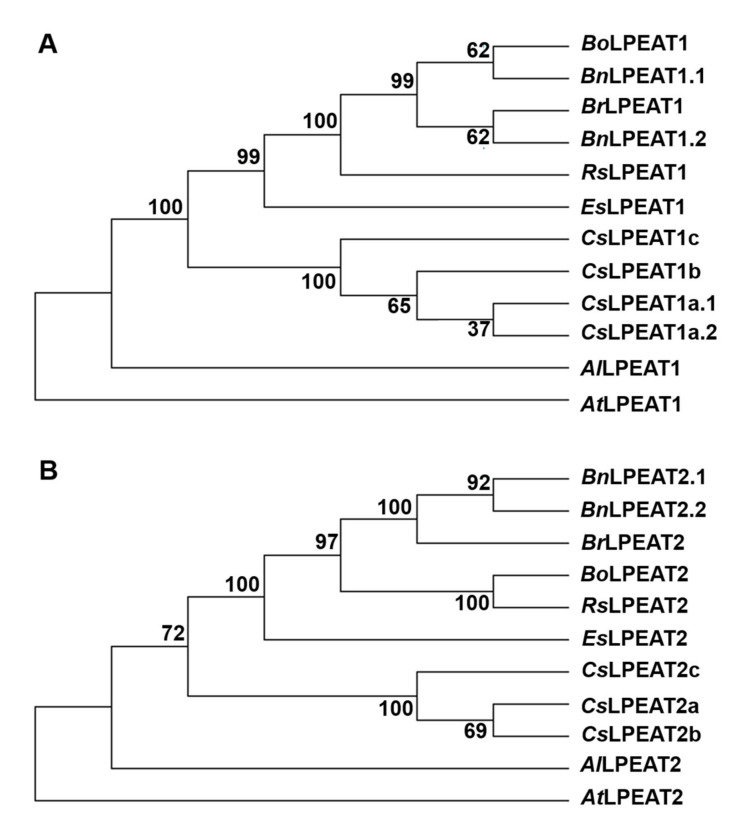
Predicted evolutionary relations between *Brassicaceae* family LPEATs. *Cs*LPEAT1 isoforms and other LPEAT1 plant amino acid sequences are analyzed in panel (**A**), while *Cs*LPEAT2 isoforms and other LPEAT2 plant amino acid sequences are analyzed in panel (**B**). Both trees are rooted in the corresponding Arabidopsis thaliana (At) orthologues (*At*LPEAT1 for panel A, *At*LPEAT2 for panel (**B**)). All gene denotations and their species of origin are elaborated on in Supplementary Table S4.

Hexaploid *Camelina sativa* genome constitutes of three merged subgenomes. Two of them are a result of an auto-hybridization event between *Camelina neglecta*-like plants. The third subgenome appeared through hybridization of the aforementioned auto-allotetraploid and *Camelina hispida* [29]. We conducted evolutionary analyses of the amplified *Cs*LPEAT isoforms (as well as of reference *Cs*LPEAT1b and *Cs*LPEAT1c) and other LPEATs of *Brassicaceae* family (Figure 4). Both generated *Cs*LPEAT1 and *Cs*LPEAT2 trees revealed that most likely *Cs*LPEAT1c and *Cs*LPEAT2c are isoforms belonging to the *Camelina hispida*-originated subgenome, since they are more distantly related to the other two isoforms of their respective *Cs*LPEAT.

A conserved domain search [30] of the *Cs*LPEAT protein sequences, showed that all isoforms possessed LPE-binding domains. Additionally, *Cs*LPEAT2 isoforms all had calcium-binding domains, characteristic of proteins participating in calcium cell signaling pathways (Supplementary Figures S3 and S4). Biochemical assays estimating the effects of calcium ions on activity of microsomal fraction LPEATs of *C. sativa* seeds, however, showed that the addition of these ions to the reaction mixture does not stimulate enzyme activity and even showed some inhibitory effects [26]. Thus, the assays performed in this study were not supplemented with calcium ions. Amino acid sequence comparisons revealed only minor differences between the sequences; the lowest similarity was observed between *Cs*LPEAT2a and *Cs*LPEAT2c and between *Cs*LPEAT2b and *Cs*LPEAT2c (which have predicted distinct origin). Protein sequences of *Cs*LPEAT1a.1 and *Cs*LPEAT1a.2 showed differences only in three amino acid positions. One of them indicted a replacement of phenylalanine to cysteine near the active site, in the area where LPE-binding domain is present (Supplementary Table S1; Supplementary Figures S3 and S4).

### 2.3. CsLPEAT1 and CsLPEAT2 Genes Expression Differs between Plant Tissues

**Figure 5:**
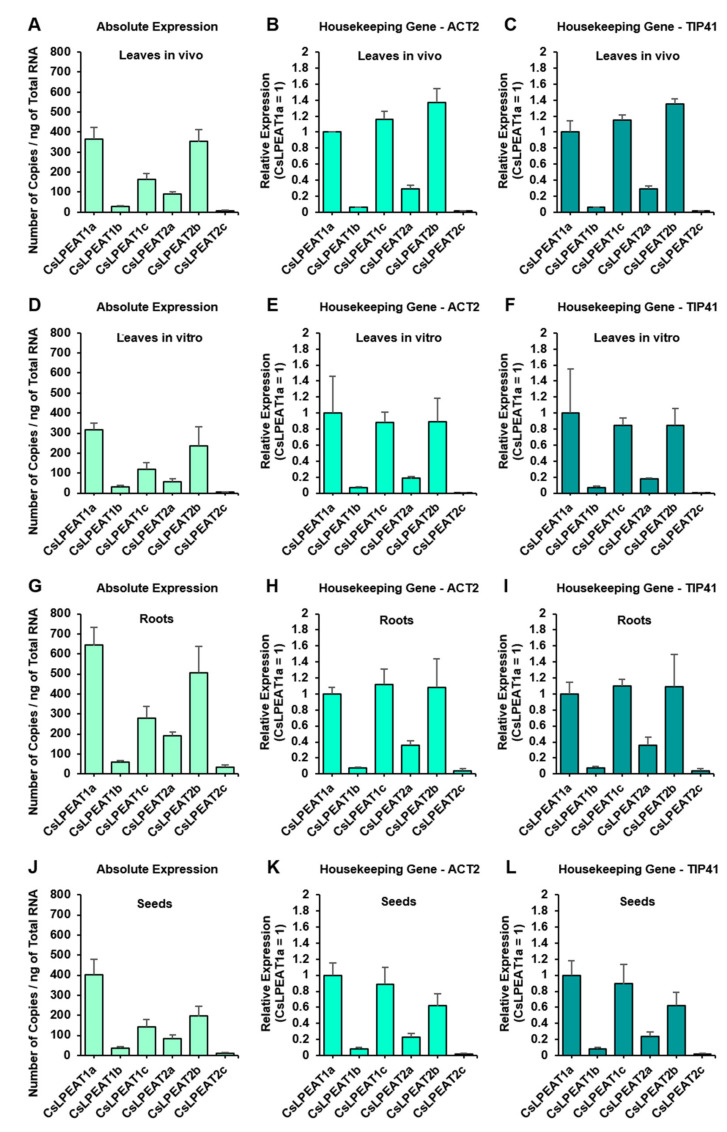
Analysis of differences in *Cs*LPEAT1 and *Cs*LPEAT2 isoform expression between different plant tissues: in vivo leaves (**A**–**C**), in vitro leaves (**D**–**F**), in vitro roots (**G**–**I**) and seeds (**J**–**L**). Panels (**A**,**D**,**G**,**J**) represent absolute quantification expression measurements, while other panels represent relative expression in comparison to *Cs*ACT2 (**B**,**E**,**H**,**K**) and *Cs*TIP41 (**C**,**F**,**I**,**L**). Error bars indicate standard deviations (SD) between biological replicates (*n* = 3).

In order to determine the expression patterns of all six *Cs*LPEAT1 and *Cs*LPEAT2 isoforms in various plant tissues, we conducted both absolute (Figure 5A,D,G,J) and relative (Figure 5B,C,E,F,H–J,L) expression analyses. In case of most isoforms, patterns of absolute and relative expression were similar to each other, except for *Cs*LPEAT1c, whose expression seemed to be higher, when determined utilizing relative quantifications. In most cases *Cs*LPEAT1a (corresponding to its predicted variants *Cs*LPEAT1a.1 and *Cs*LPEAT1a.2) was the highest expressed *Cs*LPEAT1 isoform, and *Cs*LPEAT2b was the highest expressed *Cs*LPEAT2 isoform. *Cs*LPEAT1b was the least expressed *Cs*LPEAT1 isoform in all the tissues, and *Cs*LPEAT2c was the least expressed *Cs*LPEAT2 isoform. What is clear from the obtained expression patterns is the substantially higher ratio of expression of all *Cs*LPEAT1 isoforms over all *Cs*LPEAT2 isoforms in seeds, in comparison to other tissues. *Cs*LPEAT2b, the highest expressed *Cs*LPEAT2 isoform, is the dominant isoform out of both *Cs*LPEAT1 and *Cs*LPEAT2 in in vivo leave tissue, especially when we only consider relative expression. Regarding only absolute expression, all *Cs*LPEAT1 and *Cs*LPEAT2 isoforms are most abundant in root tissue. Roots for this experiment were cultivated in vitro, as was leaves’ tissue analyzed in panels D, E, and F. In comparison to *Cs*LPEATs expression in in vitro leaves, *Cs*LPEATs expression in in vitro roots is twice as high.

### 2.4. Diverse Acyl-CoA Utilization by CsLPEAT Enzymes at Various Temperatures

The sequences of the five discovered *Cs*LPEAT genes were expressed in a haploid knock-out mutants of *ΔALE* yeast strain deficient in PE synthesis via LPLAT activity, to determine their properties and activity at different temperatures. The transformation revealed that all *Cs*LPEAT enzymes/isoforms were able to complement this type of deficiency of PE synthesis in the utilized *ΔALE* mutant (Supplementary Figure S5).

**Figure 6:**
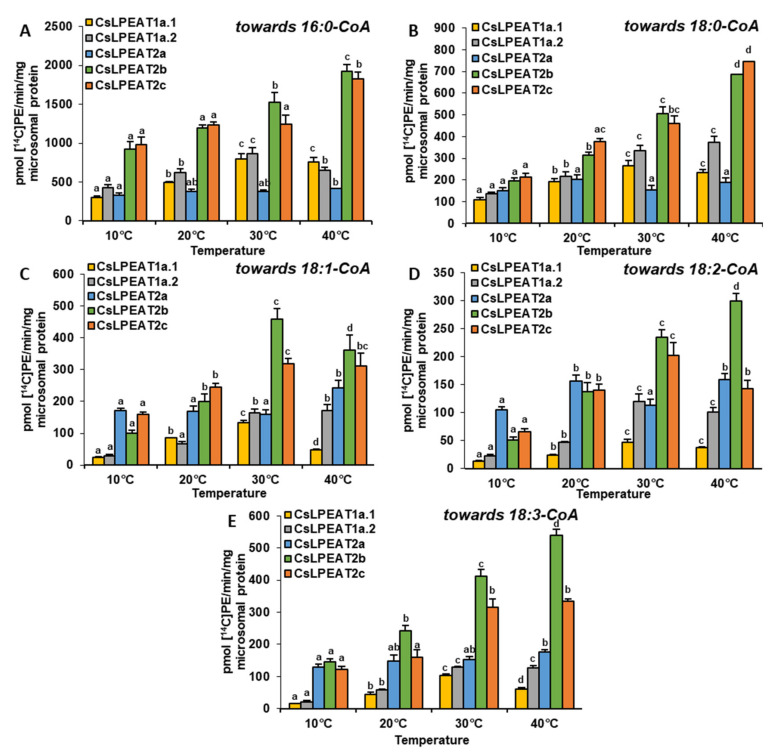
Acyl-CoA specificities of *Cs*LPEAT isoforms with various acyl-CoA donors and 18:1-LPE as an acyl acceptor, in a yeast system. Each panel corresponds to activity towards a different acyl donor: (**A**)—16:0-CoA, (**B**)—18:0-CoA, (**C**)—18:1-CoA, (**D**)—18:2-CoA and (**E**)—18:3-CoA. Error bars indicate the SD between at least three repeats (*n* ≥ 3). Statistical analysis of the difference in activity of each isoform at various tested temperatures were done by one-way ANOVA followed by Tukeys test. Different letters indicate significant difference (*p* ≤ 0.05).

**Figure 7:**
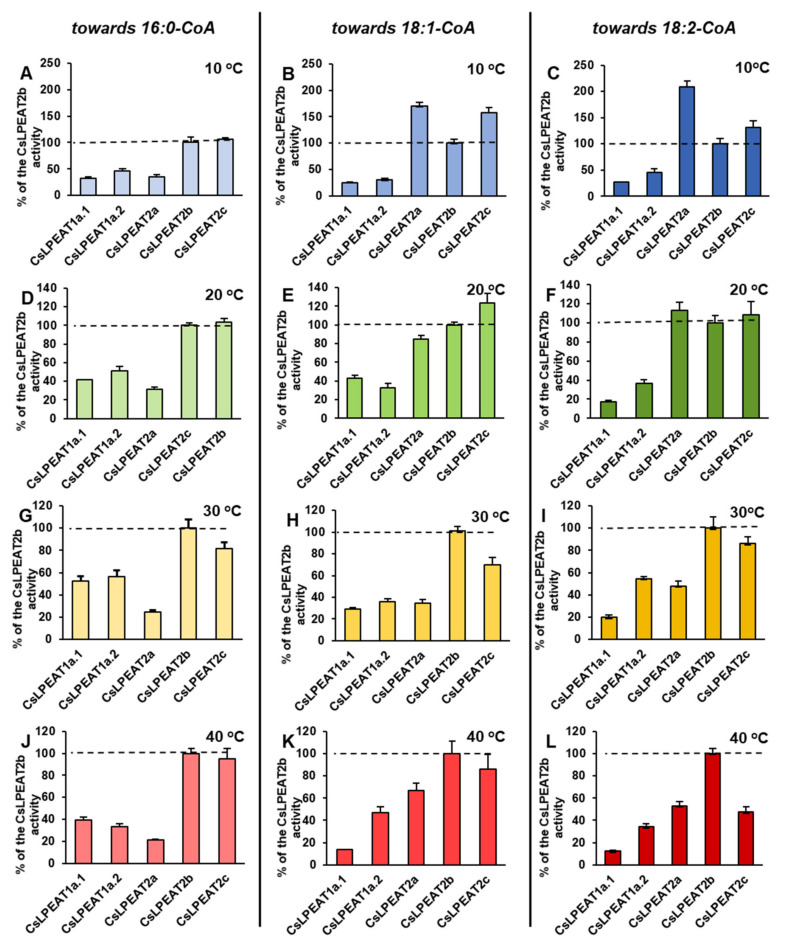
Preferences towards different acyl donors of *Cs*LPEAT isoforms at different temperatures. Activities of *Cs*LPEAT isoforms are presented in corresponding panel: towards 16:0-CoA (**A**,**D**,**G**,**J**), towards 18:1-CoA (**B**,**E**,**H**,**K**), towards 18:2-CoA (**C**,**F**,**I**,**L**). Activities are expressed as percentages of *Cs*LPEAT2b activity with a particular acyl-CoA. The data presented in Figure 6 were used for the calculations. Error bars indicate the SD between at least three repeats (*n* ≥ 3).

**Figure 8:**
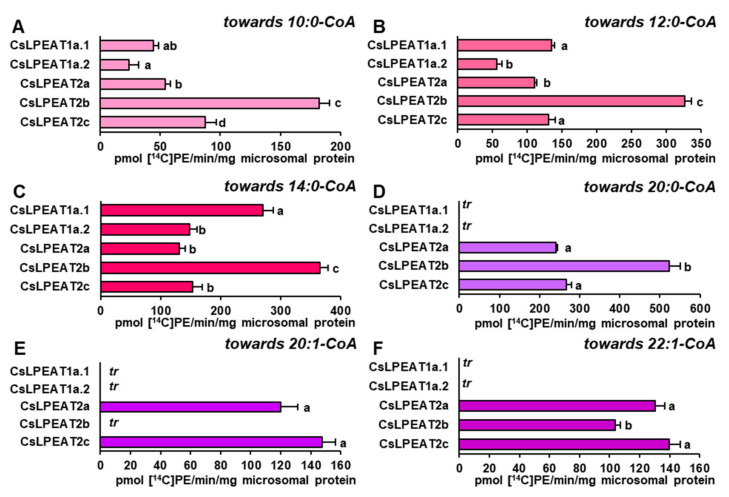
Acyl-CoA specificity of *Cs*LPEAT isoforms towards short and very long chain acyl-CoA donors and 18:1-LPE as an acyl acceptor, in yeast system. Panel (**A**–**C**) correspond to medium-chain fatty acids (10:0-CoA, 12:0-CoA and 14:0-CoA, respectively), and panel (**D**–**F**) toward VLCFA (20:0-CoA, 20:1-CoA and 22:1-CoA, respectively). Error bars indicate the SD between at least three repeats (*n* ≥ 3). Statistical analysis of the difference in activity of each isoform at various tested temperatures were done by one-way ANOVA followed by Tukey’s test. Different letters indicate significant difference (*p* ≤ 0.05).

After confirming their ability to synthesize PE via LPEAT action, the assays’ conditions were optimized to define their properties in in vitro enzymatic reactions. Each of the tested *Cs*LPEAT isoforms exhibited distinct preferences for the amount of microsomal fraction and reaction time (Supplementary Figures S6 and S7). The obtained results were applied in further analyses, where activity towards five acyl-CoAs at different temperatures was determined, utilizing 18:1-LPE as an acceptor of acyl groups (Figure 6). Moreover, we did a percentage estimation for the most preferred acyl donor (16:0-CoA) and two other acyl donors—ones which showed the biggest variability (18:1-CoA and 18:2-CoA). The activities of the enzymes were presented as the percentage of their respective *Cs*LPEAT2b activity—*Cs*LPEAT2b was treated as reference (Figure 7 and Figure 8).

The obtained results showed that 16:0-CoA was the most favored, by all *Cs*LPEAT enzymes, fatty acid donor at each tested temperature. Variants of *Cs*LPEAT1 enzymes reached the highest activity towards this acyl group at 30 °C. *Cs*LPEAT1a activity increased about 2.7 times (up to 798 pmol of de novo synthesized PE/min/mg microsomal protein), whereas *Cs*LPEAT1b increased about 2.0 times (up to 861 pmol of de novo synthesized PE/min/mg microsomal protein) compared to the lowest activity, detected at 10 °C. A further temperature increase from 30 to 40 °C reduced the activity by 6 and 25% for *Cs*LPEAT1a.1 and *Cs*LPEAT1a.2, respectively. *Cs*LPEAT1 enzymes were not the most active in comparison to the other tested *Cs*LPEAT enzymes. They obtained approximately 52–56% of maximum *Cs*LPEAT2b activity at 30 °C. The *Cs*LPEAT2b and *Cs*LPEAT2c peaks of activity were detected at 40 °C. They doubled their lowest activity at 10 °C and reached the activity of approximately 1900 pmol of de novo synthesized PE /min/mg microsomal protein, which makes them the most active among all tested *Cs*LPEAT enzymes. The *Cs*LPEAT2a variant exhibited a rather stable preference, regardless of temperature changes; their activity increased only slightly between 10 °C and 40 °C. This variant also revealed the lowest activity towards 16:0-CoA among all tested *Cs*LPEATs; at 10 °C it reached 35% and parallel with increasing temperature its activity declined to 21% of *Cs*LPEAT2b activity (Figure 6A; Figure 7A,D,G,J).

The second tested acyl group, 18:0-CoA, was primarily favored by *Cs*LPEAT2c enzyme, regardless of temperature (except for 30 °C, at which temperature 18:0-CoA was a bit better accepted by the *Cs*LPEAT2b isoform). The *Cs*LPEAT2c activity with 18:0-CoA gradually raised with temperature up to 40 °C, reaching activity equal to 745 pmol of de novo synthesized PE/min/mg microsomal protein, 3.5 times greater than at 10 °C. *Cs*LPEAT2b and *Cs*LPEAT1a.2 also elevated their activity towards 18:0-CoA parallel with increasing temperature, attaining 346% and 275% of the activity detected at minimal temperature. The activity of *Cs*LPEAT1a.1 was amplified 2.5 times between 10 °C and 30 °C. Further increase in temperature only marginally decreased *Cs*LPEAT1a.1 activity. Similarly, to its activity towards 16:0-CoA, *Cs*LPEAT2a did not exhibit any significant changes in preference towards 18:0-CoA due to temperature changes. Its activity fluctuated between 151 and 204 pmol of de novo synthesized PE/min/mg microsomal protein (Figure 6B).

The activity of analyzed enzymes towards 18:1-CoA was distinguished by the greatest variability. At 10 °C, 18:1-CoA was very efficiently utilized by *Cs*LPEAT2a and *Cs*LPEAT2c, what amounted to 171 and 158 pmol of de novo synthesized PE/min/mg microsomal protein. These isoforms have been 70% and 58% more active than the *Cs*LPEAT2b reference. *Cs*LPEAT2a maintained a rather stable activity for most temperatures, a maximum value obtained at 40 °C, which was equal to 242 pmol of de novo synthesized PE/min/mg microsomal protein. Regarding *Cs*LPEAT2c enzyme, its activity at 20 °C dominated over the rest of the tested isoforms. Compared to relative activity of the chosen reference its activity was 24% higher and constituted about 246 pmol of de novo synthesized PE/min/mg microsomal protein. The increased temperature favored the action of *Cs*LPEAT2b, especially at 30 °C when *Cs*LPEAT2b activity reached 460 pmol of de novo synthesized PE/min/mg microsomal protein—which was *Cs*LPEAT2b maximum activity. At 40 °C the isoform’s activity declined by 22%, however, it remained the most active variant towards 18:1-CoA. The participation in utilization of 18:1-CoA by *Cs*LPEAT1 enzymes was rather modest. *Cs*LPEAT1a.1 was most active at 30 °C. Further increase of temperature dramatically decreased utilization of this acyl donor by 65% compared to the maximum. In case of *Cs*LPEAT1a.2, its activity was higher at higher temperatures and attained the peak of its activity at 40 °C. The relative activity compared to the reference variant was the highest at 20 °C (43% of reference activity) and at 40 °C (47% of reference activity) for *Cs*LPEAT1a.1 and *Cs*LPEAT1a.2, respectively (Figure 6C; Figure 7B,E,H,K).

Similar, to the above described utilization of 18:1-CoA, the utilization of 18:2-CoA also varied depending on the temperature and enzymes. Nevertheless, this acyl donor was the least accepted one. At 10 and 20 °C, *Cs*LPEAT2a was more active than other tested enzymes with 18:2-CoA. Its activity attained 104 and 150 pmol of de novo synthesized PE/min/mg microsomal protein, and showed 107% and 13% greater activity than reference, respectively, at those temperatures. At higher temperatures, *Cs*LPEAT2a activity with 18:2-CoA fluctuated between 112 and 158 pmol of de novo synthesized PE/min/mg microsomal protein, finally obtaining about 50% of reference activity. The second variant most active with 18:2-CoA at the range of 10–30 °C was *Cs*LPEAT2c. Its activity progressively increased from 66 to 202 pmol of de novo synthesized PE/min/mg microsomal protein. *Cs*LPEAT2c activity towards 18:2-CoA started (in this range of temperatures) from activity 31% higher and ending with activity 15% lower than reference. At 40 °C relative activity of *Cs*LPEAT2c with 18:2-CoA decreased to about 50% of the reference one. The *Cs*LPEAT1 enzymes again exhibited the lowest activity towards 18:2-CoA. Both reached peak activity at 30 °C: 47 and 119 pmol of de novo synthesized PE/min/mg microsomal protein for *Cs*LPEAT1a and *Cs*LPEAT1b, respectively. Their relative activity was the highest at 30 °C reaching approximately 10 and 55% of reference activity with 18:2-CoA, respectively for *Cs*LPEAT1a and *Cs*LPEAT1b (Figure 6D; Figure 7C,F,I,L).

The activity of all tested enzymes towards 18:3-CoA revealed strong efficiency of utilization of this acyl donor by *Cs*LPEAT2 enzymes. Parallel with rising temperature activity of *Cs*LPEAT2a and *Cs*LPEAT2b increased 2.7 and 3.7 times, respectively. *Cs*LPEAT2a activity, similar to other acyl groups, did not differ significantly regardless of temperature. It maintained efficiently utilizing 18:3-CoA at similar levels, which oscillated between 128 and 175 pmol of de novo synthesized PE/min/mg microsomal protein. The activity of *Cs*LPEAT1 enzymes towards 18:3-CoA was rather negligible at 10 °C. At higher temperatures *Cs*LPEAT1a.1 efficiency with 18:3-CoA slowly enhanced, reaching maximum at 30 °C and then being reduced by 40% at 40 °C. *Cs*LPEAT1a.2 activity with 18:3-CoA also increased and reached a constant at 30 and 40 °C—125 pmol of de novo synthesized PE/min/mg microsomal protein (Figure 6E).

### 2.5. CsLPEAT2 Isoforms Are Specific towards Saturated and Very-Long-Chain Fatty Acids

Considering the possible availability of short and very-long-chain fatty acids in acyl-CoA pool and to determine fringe specificities of each *Cs*LPEATs, their preferences towards these acyl groups were examined. Reactions were conducted at 30 °C and 18:1-LPE was chosen as an acyl acceptor (Figure 8).

Short, saturated acyl-CoA were utilized by all enzymes. The highest specificity was detected towards 14:0-CoA, especially by *Cs*LPEAT2b and *Cs*LPEAT1a.1 enzymes, which activity amounted to 365 and 270 pmol of de novo synthesized PE/min/mg microsomal protein. The other tested enzymes utilized 14:0-CoA with similar efficiency, which amounted to approximately 132–150 pmol of de novo synthesized PE /min/mg microsomal protein (Figure 8C). All tested enzymes were also active with 12:0-CoA and 10:0-CoA with similar preference pattern towards both. The lowest activity was detected for *Cs*LPEAT1a.2, whereas *Cs*LPEAT2b was the most active isoform with those acyl-CoA. *Cs*LPEAT1a.2 activity with 12:0-CoA amounted to 56 pmol of de novo synthesized PE/min/mg microsomal protein and *Cs*LPEAT2b activity with 12:0-CoA amounted to 328 pmol of de novo synthesized PE/min/mg microsomal protein. Both activities were twice higher than the corresponding activities with 10:0-CoA. The activity of other enzymes declined in similar manner with 10:0-CoA. Only *Cs*LPEAT1a.1 activity with 10:0-CoA (the second most active variant with 12:0-CoA) was reduced not two but three times (Figure 8A–C).

**Figure 9:**
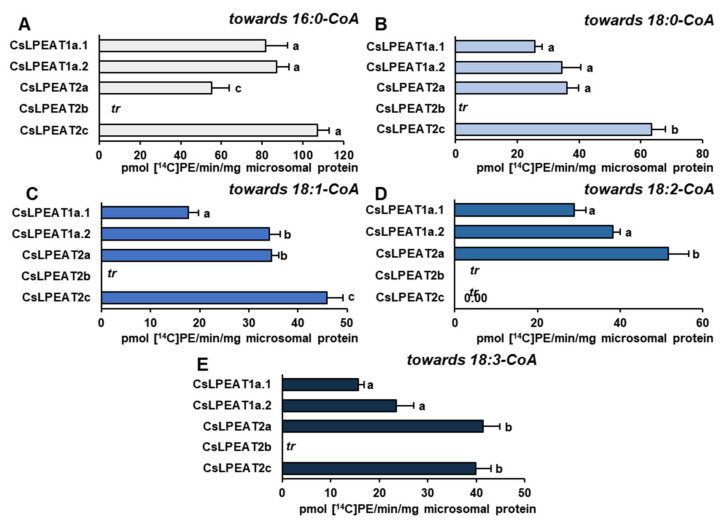
Acyl-CoA specificities of *Cs*LPEAT isoforms with various acyl-CoA donors and 16:0-LPE as an acyl acceptor, in a yeast system: (**A**)—towards 16:0-CoA, (**B**)—towards 18:0-CoA, (**C**)—towards 18:1-CoA, (**D**)—towards 18:2-CoA and (**E**)—towards 18:3-CoA. Error bars indicate the SD between at least three repeats (*n* ≥ 3). Statistical analysis of the difference in activity of each isoforms at various tested temperature were done by one-way ANOVA followed by Tukeys test. Different letters indicate significant difference (*p* ≤ 0.05).

On the other hand, acyl donors with very-long-chain fatty acids were accepted only by *Cs*LPEAT2 enzymes. The highest preference was detected towards 20:0-CoA. Additionally, for *Cs*LPEAT2a and *Cs*LPEAT2b, it was the second most favored acyl donor. Their activity with this acyl-CoA amounted to 523 and 241 pmol of de novo synthesized PE/min/mg microsomal protein, which constituted 65% and 35% of their activity towards 16:0-CoA, respectively. *Cs*LPEAT2c activity was similar to its specificity towards 18C unsaturated fatty acids and amounted to about 266 pmol of de novo synthesized PE/min/mg microsomal protein, being the second most active variant towards 20:0-CoA (Figure 8D). Two other very-long-chain acyl donors were utilized with similar efficiency (Figure 9E,F). *Cs*LPEAT2c enzymes revealed a little bit higher activity towards 20:1-CoA and 22:1-CoA, reaching 148 and 140 pmol of de novo synthesized PE/min/mg microsomal protein, respectively. *Cs*LPEAT2a enzymes activity was reduced by 20% and 8% compared to *Cs*LPEAT2c activity towards 20:1-CoA and 22:1-CoA, respectively. *Cs*LPEAT2b utilized 22:1-CoA to a lesser extent than the two other *Cs*LPEAT2 isoforms, reaching activity equal to 104 pmol/min/mg microsomal protein. *Cs*LPEAT2b specificity towards 20:1-CoA was poor and only trace levels of de novo synthesized PE could be detected (Figure 8D–F).

### 2.6. Distinct Ability or Inability to Utilize Various Acyl Acceptors by CsLPEAT Enzymes

Based on previous tests describing fatty acids composition of *sn-1* position of PE of *C. sativa* leaves, assays determining *Cs*LPEAT activity and substrate specificity with different acyl acceptors such as 16:0-LPE and 18:2-LPE were conducted. Again, five most abundant fatty acids present in PE pool of different tissues were chosen. Assays with 18:1-LPE were described in previous section.

**Figure 10:**
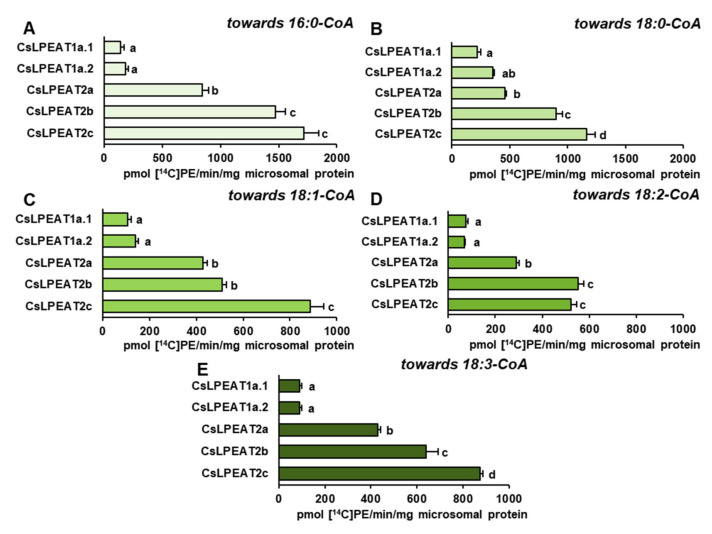
Acyl-CoA specificities of *Cs*LPEAT isoforms with various acyl-CoA donors and 18:2-LPE as an acyl acceptor, in a yeast system: (**A**)—towards 16:0-CoA, (**B**)—towards 18:0-CoA, (**C**)—towards 18:1-CoA, (**D**)—towards 18:2-CoA and (**E**)—towards 18:3-CoA. Error bars indicate the SD between at least three repeats (*n* ≥ 3). Statistical analysis of the difference in activity of each isoform at various tested temperatures were done by one-way ANOVA followed by Tukeys test. Different letters indicate significant difference (*p* ≤ 0.05).

Despite the predominant presence of 16:0 at *sn-1* position, the activity of *Cs*LPEAT enzymes towards 16:0-LPE was much smaller compared to other tested LPE. *Cs*LPEAT2b even exhibited no activity (on the detection level) towards this acyl acceptor. The tested enzymes preferred 16:0-CoA as an acyl donor, especially *Cs*LPEAT2c, followed by *Cs*LPEAT1a.2 and 1a.1, and by *Cs*LPEAT2a: 107, 87, 82 and 55 pmol of de novo synthesized PE/min/mg protein, respectively (Figure 9A). The 18:0-CoA and 18:1-CoA were utilized in a comparable manner. *Cs*LPEAT2c exhibited the highest preference, and its activity amounted to approximately 63 and 45 pmol of de novo synthesized PE/min/mg protein, respectively. The *Cs*LPEAT1a.2 and *Cs*LPEAT2a showed the same activity towards both acyl donors in the range of 34–36 pmol of de novo synthesized PE/min/mg protein. The second to last active variant was *Cs*LPEAT1a.1. Its activity towards both acyl-donors reached only 40% of maximum activity exhibited by *Cs*LPEAT2c (Figure 9B,C). Towards 16:0-LPE and 18:2-CoA only *Cs*LPEAT2a, *Cs*LPEAT1a.2 and *Cs*LPEAT1a.1 were functional, in this order, reaching 51, 38 and 29 pmol of de novo synthesized PE/min/mg protein of activity (Figure 9D). Towards 18:3-CoA, the highest preference was shown by *Cs*LPEAT2a. Other enzymes (except non-active *Cs*LPEAT2b) reached 97 (*Cs*LPEAT2c), 57 (*Cs*LPEAT1a.2) and 38% (*Cs*LPEAT1a.1) of its activity (Figure 9E). The third tested acyl acceptor was 18:2-LPE. Linolenic acid is the third most abundant fatty acid present in *sn-1* position of PE in leaves, it constitutes approximately 13% of all the fatty acids at that stereospecific position. In assays with this acyl acceptor, 16:0-CoA was most efficiently utilized by all *Cs*LPEAT2 enzymes reaching 1718, 1475 and 856 pmol of de novo synthesized PE/min/ mg protein for isoforms 2c, 2b, and 2a, respectively. *Cs*LPEAT1a variants utilized this acyl donor on significantly lower level, only 8 and 11% of maximum activity towards this fatty acid for *Cs*LPEAT1a.1 and *Cs*LPEAT1a.2, respectively (Figure 10A). Both 1a.1 and 1a.2 variants exhibited the highest preference towards 18:0-CoA, which accounted for respectively about 19 and 30% of maximum activity towards 18:0-CoA. Again, *Cs*LPEAT2c was the most active enzyme, followed by 2b and 2a isoforms reaching in order 1162, 899 and 460 pmol of de novo synthesized PE/min/mg protein (Figure 10B). Variants of *Cs*LPEAT1a exhibited quite similar activity towards unsaturated fatty acids donors: towards 18:1-CoA 106-142, towards 18:2-CoA 69-76 and towards 18:3-CoA 89-91 pmol of de novo synthesized PE/min/mg protein. Among the *Cs*LPEAT2 isoforms 18:1-CoA was best utilized in assays with 18:2-LPE by isoform 2c, followed by 2b and then 2a. Those activities amounted to 887, 511 and 430 pmol of de novo synthesized PE/min/mg protein, respectively. Similar values were observed towards 18:3-CoA: 874, 640 and 430 pmol PE/min/mg protein, respectively. A different manner of preference was observed towards 18:2-CoA, where the best utilization efficiency was shown by *Cs*LPEAT2b, followed by *Cs*LPEAT2c and then by *Cs*LPEAT2a. *Cs*LPEAT2b activity in assays with 18:2-LPE and 18:2-CoA accounted for 553 pmol of de novo synthesized PE/min/mg protein, whereas two other enzymes exhibited 94% (*Cs*LPEAT2c) and 52% (*Cs*LPEAT2a) of that activity (Figure 10C–E).

## 3. Discussion

### 3.1. Acyl-CoA: Lysophosphatidyethnolamine Acytransferases Present in Vegetative and Generative Organs of C. sativa Respond Directly to Temperature Changes and Shape Phospholipids’ Composition

In our experiments, chill temperature promoted LPEAT enzymes substrate specificity towards 18:3-CoA. The reverse affinity was observed at higher temperatures, greater than 30 °C, where declining specificity towards 18:3-CoA and raising specificity towards monounsaturated 18:1-CoA were noticed. These preferences correlate with the fatty acid composition of acyl-lipids present in plants subjected to altered temperatures. A previous study on oily plants, grown in higher temperatures, revealed that seeds of such plants produced decreased levels of 18:2 and 18:3, and increased of 18:1 content [31,32,33]. Studies conducted on *A. thaliana* showed that leaf membrane lipid composition was characterized by a decrease in levels of 18:3 and 16:3, and elevated levels of 16:0 and 18:1. Leaf tissue exposed to heat stress also possessed higher amounts of 18:2, in contrast to the seeds [34,35,36,37]. In our study, similar preference towards 18:2-CoA was observed in case of LPEAT enzymes present in vegetative organs. It was similarly not observed in seed tissue. The *C. sativa* in vivo leaves were the only studied tissue with strong preference towards 16:0-CoA at high temperature. This preference may be an adaptation to heat stress to maintain proper, solid membrane structure in tissues most exposed to environmental conditions [35]).

Previously observed changes also concerned the PE pool, in which the amount and composition of fatty acids adjusted to the temperature. The studies, conducted at temperatures below 5 °C, revealed that levels of PE in *Brassica napus* and in *A. thaliana* leaves were elevated and characterized by lipid molecular species possessing linoleic acid [34,38]. On the contrary, heat-stressed plants exhibited significantly reduced levels of PE and reduced amounts of 18:3. Furthermore, amounts of oleic acid increased in all molecular species [37]. The possible changes of PE composition are consistent with LPEATs preferences, which may play a dominant role in their regulation, especially its affinity towards 18:1-CoA and 18:3-CoA at various temperatures. It has been previously proven that trienoic fatty acids boosted plant tolerance in colder temperatures, while its diminishing amount at higher temperatures influenced better acclimatization [39].

Phosphatidylethanolamine is one of the most abundant phospholipids in plant tissues. The observed correlation of LPEAT activity with different tissues acyl composition and PE pool may arise not only from its physiological role in vegetative tissues. PE was also shown as a major substrate for PDAT enzyme in vitro activity (TAG production). If PE is a substrate for PDAT in vivo its acyl profile may influence the final oilseed plants seed composition [17]. The observed, higher amounts of 18:3 in PE pool after cold stress correlated with elevated expression of PDAT1-A, present in *C. sativa* seeds, cultivated at low temperature. PDAT1 possesses a high preference towards this fatty acid [40].

The highest activity towards each of the tested acyl donors was observed at 30 °C. The same temperature optimum was also noticed for other LPLAT enzymes present in *C. sativa* [26,28]. Declined activity was mainly observed at 10 °C and 40 °C. In those thermal conditions, the detrimental effects of temperature on protein may appear, leading in cold to inhibition of enzymatic activity, and in heat to inactivation of protein by gradual denaturation [1].

The observed distinction in acyl donor preference, in some way was also proven by competence assays conducted for microsomal fractions derived from leaves, which exhibited the most fluctuating preferences and activities due to temperatures in our study. Assays were conducted with addition of BSA, which mimi*Cs* the activity of ACBP present in vivo. In in vitro conditions BSA, similarly to ACBP in vivo, can bind acyl-CoAs and reduce availability of free acyl-CoAs for the acyltransferases, imitating natural conditions [41,42]. In both temperatures (10 and 30 °C), 18:1-CoA was better utilized than 18:3-CoA, however the biggest dissimilarities were observed at 30 °C, where 18:3-CoA was accepted at least 3 times worse than 18:1-CoA. At elevated temperatures, LPEATs present in vitro leaves also exhibited higher relative preference towards 16:0-CoA, which was not previously noticed in substrate specificity tests. In such assays, however, the elevated absolute activity for 16:0-CoA increased up to 30 °C. Observed preferences, which are not fully correlated with observed preferences towards single acyl donors, demonstrate that in acylation of LPE in in vivo conditions, substrate specificities of LPEAT enzymes are not the only factor, which matters. The availability of individual acyl-CoA in cytosolic acyl-CoA pool also plays an important role. The main source of acyl-CoA in this pool are plastids where fatty acids are synthesized. Plastids enrich cytosolic pool of acyl-CoA mainly with 18:1-CoA and in smaller amounts with 16:0-CoA and 18:0-CoA. The composition of cytosolic acyl-CoA pool can be regulated also by reverse reaction of LPLAT enzymes, especially by LPCAT, which supplements this pool in unsaturated fatty acids by transferring modified fatty acids from PC, major place of desaturase activity [28,43] (Li-Beisson et al. 2013, Klińska et al. 2019). Based on Ruiz-Lopez et al.’s [44] study, the dominant 18C acyl-CoA in cytosolic acyl-CoA pool is 18:1-CoA, towards which, in our competence assay, microsomal LPEAT enzymes exhibited the highest preference. It was also the best utilized acyl donor in assays determining the intensity of PE remodeling. These assays were conducted for leaves in vivo microsomal fractions, at two different temperatures to estimate how efficient the remodeling process is in tissues most exposed to ambient temperatures. We estimated the potential time of complete fatty acid turnover in PE pool. Calculations were made for remodeling of the *sn-1* and *sn-2* positions simultaneously, but we must remember that fatty acids from *sn-2* position are most preferred in acyl editing process and complete exchange of this position can be shorter. The observed remodeling intensity was highly dependent on temperature. At 30 °C its intensity was about 3 to about 5 times faster (depending on acyl-CoA added to the assays) than at 10 °C. The dependence of remodeling intensity on a kind of acyl-CoA present in the assay was a bit surprising as the participation of backward LPEAT reaction in this process was rather small (data not presented). Thus, this data suggests, that the kind of acyl-CoA present in the assays effects the formation of LPE, e.g., via lipase or PDAT activity and in this way affects remodeling intensity. However, so far there is no information about this type of effects caused by acyl-CoA and this issue needs further study. Of the tested acyl-CoA 18:1-CoA had the highest effect on such a reaction and 18:3-CoA had the lowest effect. However, at 30 °C 18:2-CoA was even more effective than 18:1-CoA.

The physiological importance of the observed acyl-CoA effect on lysophospholipid formation is hard to explain in regards to PE. It is possible that it is a common phenomenon, similar for all phospholipids. In our previous study, we have observed similar feature of the PC [28]. In in vitro tests utilizing *C. sativa* microsomal fractions, the rate of PC remodeling was the highest, when the reaction mixture included 18:1-CoA, lower when it included 18:2-CoA, and slowest with 18:3-CoA. Plastid-derived 18:1 must be desaturated on PC to produce 18:2 and 18:3. Thus, the stimulating effect of 18:1-CoA on LPC production could have an important physiological significance. Equivalent physiological importance of higher 18:1-CoA incorporation into PE needs further investigation.

The tested LPEATs are enzymes involved in the synthesis of [^14^C]PE from LPE and [^14^C]acyl-CoA. However, PE and LPE (via reverse reaction) can also be synthesized by other LPLATs [20]. Consequently, we cannot measure the exact activity of LPEATs in our assays with microsomal fractions derived from plant tissues. Nevertheless, based on previously demonstrated substrate specificity [26,28] as well as based on the presented temperature-dependent substrate specificity, we can certainly assume that LPEAT enzymes play the leading role in PE production via LPLAT action. The role in temperature sensing for LPCAT enzymes, which can also acylate LPE, was rather poor; temperature alterations did not influence any substrate preferences of LPCAT enzymes present in leaves and roots.

### 3.2. The Origin of the CsLPEAT Isoforms, and Their Diverse Expression Pattern in C. sativa Organs

*C. sativa* is a hexaploid organism, which originated from two genome hybridization events. First hybridization event occurred between members of two organisms, both possessing a *Camelina neglecta*-like genome, leading to generation of an auto-allotertaploid (4n). The second hybridization happened interspecies between the auto-allotetraploid and *Camelina hispida*. As a result, *C. sativa* is comprised of three stable subgenomes [29]. On account of the high complexity of Camelina genome, most genes are present in three homeolog variants. LPEAT1 and LPEAT2 genes also have three homeologs each in *C.sativa*. Based on bioinformati*Cs* analyses we successfully amplified 5 different isoforms of *Cs*LPEATs. Three of them had sequences highly similar to each *Cs*LPEAT2 homeologs, present in the database of predicted *C. sativa* DH55 cultivar genes annotation. We named them: *Cs*LPEAT2a, *Cs*LPEAT2b, and *Cs*LPEAT2c. In case of two other found sequences, the analysis revealed that they are variants of one homeolog, which we called *Cs*LPEAT1a. The two variants were named accordingly *Cs*LPEAT1a.1 and *Cs*LPEAT1a.2. [45]

In the conducted evolutionary analyses, we also took into account two other *Cs*LPEAT1 isoforms, which we could not amplify from our cultivar’s cDNA (*Cs*LPEAT1b and *Cs*LPEAT1c). The reason behind our inability to amplify these homeologs lies probably in high abundance of *Cs*LPEAT1a variants in the cDNA. Regardless, the phylogenetic analysis itself revealed that both *Cs*LPEAT1c and *Cs*LPEAT2c originated from *C. hispida* genome with high probability. The other homeologs are likely derived from *C. neglecta* due to their calculated closer phylogenetic distance. Two of *Cs*LPEAT1a isoform variants are highly similar, which influenced an insignificant bootstrap value for them. *Cs*LPEAT1a variants differed only by one amino acid, however this difference was in close proximity to their respective LPE-binding domains. Phenylalanine in *Cs*LPEAT1a.1 is substituted for cysteine in *Cs*LPEAT1a.2. It is postulated that introduction of a polar sidechain, like in cysteine replacing a non-polar phenylalanine could have a significant effect on electrostatic and substrate-binding interactions [46]). The detected substitution may be the reason behind *Cs*LPEAT1a variants exhibiting divergent lysophospholipid acyltransferase activities and biochemical properties. In the case of *Cs*LPEAT2, we were able to amplify all three homeologs. Conserved domain analyses revealed that these isoforms all possessed additional calcium-binding domain, found also in *At*LPEAT2 [16]. Both generated *Cs*LPEAT1 and *Cs*LPEAT2 trees revealed that *Cs*LPEAT isoforms are more closely related to the other *Brassicaceae* LPEATs than to Arabidopsis LPLATs. 70% similarity of the annotated genes was shown between *A. thaliana* and *C. sativa* by Kagale et al. [45].

Expression patterns for all *Cs*LPEAT1 and *Cs*LPEAT2 isoforms were determined for vegetative and generative organs. Among *Cs*LPEAT1 isoforms, *Cs*LPEAT1a, corresponding to its predicted variants *Cs*LPEAT1a.1 and *Cs*LPEAT1a.2, was the highest expressed isoform, whereas among *Cs*LPEAT2 isoforms it was *Cs*LPEAT2b. The lowest expressed isoform was *Cs*LPEAT2c. Surprisingly, both mentioned *Cs*LPEAT2 isoforms exhibited quite comparable specificities, despite the lack of activity towards 16:0-LPE by *Cs*LPEAT2b. Such high similarities can be the reason for low expression of one of them. Relative expression patterns showed that *Cs*LPEAT1 isoforms were more dominant than *Cs*LPEAT2 in seeds in comparison to vegetative tissues. In vegetative tissues *Cs*LPEAT1 and *Cs*LPEAT2 expression was comparable, with leaves in vivo being the exception—*Cs*LPEAT2b was more expressed there. Such dissimilarities in expression in different tissues are probably a result of various biochemical properties of PE from different tissues and function, which specific tissue plays in plant physiology. Absolute expression indicated that these isoforms are expressed at a quite comparable level in case of leaves and seeds. Studies conducted on other LPLAT enzymes showed remarkably low expression in *C. sativa* leaves compared to seeds [47]. It is worth to mention that the highest absolute expression of LPEAT genes was in roots, in which we also observed a very high activity of *Cs*LPEAT enzymes. No correlation between expression levels and enzyme activity in in vitro leaves was noticed. In this tissue, all isoforms exhibited the lowest absolute expression, simultaneously displaying the highest activity of *Cs*LPEAT enzymes present in plant microsomal fraction. The reason why in vitro cultivation conditions favored *Cs*LPEAT enzymes activity remains to be elucidated in further studies.

### 3.3. Biochemical CharacteristiCs and Temperature Susceptibility of CsLPEAT Enzymes

Substrate specificity assays determining the impact of temperature on *Cs*LPEATs activity were done towards five chosen acyl donors, which also represent most abundant fatty acids in PE of different *C. sativa* tissues: 16:0, 18:0, 18:1, 18:2, and 18:3. An analysis of fatty acids of *sn-1* and *sn-2* positions of PE of leaves also proved the presence of these acyl groups. In *sn-1* position 16:0 dominated over other acyl groups, followed by unsaturated fatty acids. In *sn-2* position a reverse pattern was observed. A similar composition of fatty acids in *sn-1* position of PE was observed in *A. thaliana* leaves [37]. Nevertheless, the composition of molecular species of LPE in leaves constantly changed due to temperature alterations. In cold environment unsaturated fatty acids were present in LPE, whereas in heat stress, composition of fatty acids of LPE was quite similar to control conditions. 16:0-LPE was the most abundant, followed by 18:2-LPE [34,37]. In seeds composition of LPE was very varied with prevailing amounts of 16:0-LPE. The second most abundant LPE was 18:1-LPE, which in some studies even exceeded 16:0-LPE content [48,49]. Thus, the variety of molecular species of LPE depends on cultivation conditions and tissue of origin. In our assays we used 16:0-LPE, 18:1-LPE and 18:2-LPE, which presence in *C. sativa* was confirmed.

Among all tested LPEAT isoforms and used acyl donors, the most efficient utilization was observed towards palmitic and stearic acids regardless of supplemented acyl acceptors. This result is unexpected, considering that in *sn-2* position of phospholipids, created via eukaryotic pathway, the amount of saturated fatty acids is rather negligible [50]. However, in previous studies done by Stålberg et al. [16] and Jasieniecka-Gazarkiewicz et al. [24] the highest preference of *At*LPEAT towards 16:0-CoA was detected. Moreover, in our study, we observed that LPEAT enzymes present in microsomal fractions of different tissues of *C. sativa* revealed strong preference towards 16:0-CoA, especially in leaves. In the *sn-2* position of PE about 10% of palmitic acid was detected. This LPEAT property may play an important role in heat stress by producing PE molecules possessing two palmitic acids, which would allow for maintaining the proper structure of phospholipid membranes in elevated temperatures. Palmitic acid is one of the compounds stabilizing membrane fluidity and its amount was shown to be rising when plants were subjected to heat stress [35,37]. This correlation was also observed in our assays. The preference towards 16:0-CoA of all *Cs*LPEAT isoforms increased parallel with temperature.

*Cs*LPEAT preferences, presented in assays with microsomal fraction from leaves (both in vivo and in vitro), roots and seeds clearly indicate that activity towards 18:3-CoA at 40 °C decreases or is similar to the activity detected at 30 °C. The analysis conducted on microsomal fraction on yeast expressing different *Cs*LPEAT isoforms did not always exhibit the same preferences. *Cs*LPEAT1a.1 and *Cs*LPEAT1a.2 activities towards 18:3-CoA decreased at 40 °C, compared to 30 °C. In case of *Cs*LPEAT2 isoforms—*Cs*LPEAT2a and *Cs*LPEAT2c showed stable or only slightly higher activity, whereas activity of *Cs*LPEAT2b increased by 35% at 40 °C in comparison to 30 °C. Furthermore, the preferences towards 16:0-CoA where the highest in assays conducted with microsomal fractions derived from yeast, which was not the same for plant microsomes assays.

The observed discrepancies might be caused by the composition of lipid environment surrounding LPEAT enzymes, which may essentially influence their activity and substrate specificity. Also, each isoform might be expressed in a different manner at various temperatures, which could explain observed preferences of assays with microsomal fractions derived from plants.

This suggests that the results obtained from assays with yeast microsomal fractions elucidate mainly differences between the isoforms activity and substrate specificity. The potential physiological function of each isoform cannot be outright conjectured from these results. Here, the results from assays with plant microsomal fractions should be mainly considered as they showcase LPEAT activity in their native lipid environment.

VLCFA-rich PE species were so far detected in *Arabidopsis thaliana* leaves, roots and seeds [24,51,52]. In *Camelina sativa* the presence of gondoic acid (20:1) was detected, with its amount reaching 3.5% in 18 DAF seeds [48]. Our study shows, that *Cs*LPEAT2 exhibited a unique substrate specificity towards very-long-chain fatty acids (VLCFA). On the contrary, we did not observe overwhelming activity towards medium-chain, saturated fatty acids by *Cs*LPEAT1. This was also previously observed for *At*LPEAT isoforms [24]. The VLCFA are the dominant pool of acyl-CoA profile of *C. sativa* seeds [44,48]. Nevertheless, enzymes’ preference towards them was detected on a negligible level assuming that they do not participate in G3P pathway leading to TAG synthesis in *C. sativa* seeds [48,53]. TAG synthesis supplementation via LPCAT activity, which may introduce fatty acids into the PC pool was examined in a study conducted by Klińska et al. [28], where LPCAT enzymes present in *C. sativa* seeds did not exhibit any activity, in forward reaction, towards these acyl donors. All these results clearly show that there must be another key player in this process, since mature seeds of *C. sativa* possess approximately 23–28% of VLCFA [28,54]. The biochemical properties of *Cs*LPEAT2 isoforms indicate that they may be major players in transferring VLCFA to PE pool. VLCFA-rich PE could be thereafter transformed into PC pool via PEMTs activity and subsequently VLCFA could be transferred directly from *sn-2* position of PE or PC to TAG pool via PDAT enzymes [17,55,56,57]. However, in such case *C. sativa* PDAT should be extremely specific towards VLCFA as only their small amounts were detected in PE and PC of *C. sativa* seeds [26,28].

Despite the high quantity of 16:0-LPE in plants, in each case, 16:0-LPE turned out to be the worst utilized acyl acceptor by all *Cs*LPEAT isoforms. *Cs*LPEAT2c did not exhibit any activity towards 18:2-CoA, while corresponding *Cs*LPEAT2b activity equaled zero or was under the detection level also with other tested acyl-CoA. Decreased preferences towards 16:0-LPE, compared to 18:1-LPE, were previously noticed by Stålberg et al. [16] and Hishikawa et al. [58], both in case of 16:0-CoA or 18:1-CoA used as acyl donors. The biggest differences were observed for *At*LPEAT2, which was also observed in our study. Another study determining selectivity of other LPLAT enzymes—LPAAT revealed that saturated lysophospholipids are not favored by these enzymes, unlike the tested 18:1-LPE [59]. The elevated content of 16:0-LPE characteristic for leaf membrane lipid composition may be explained by such enzyme’s preference. These LPE species may be favored at elevated temperatures by *Cs*LPEAT, which may correlate with adaptation and adjustment of phospholipid membrane for this biotic stress. To prove such property further investigations are needed.

The activity of both variants of *Cs*LPEAT1a (*Cs*LPEAT1a.1 and *Cs*LPEAT1a.2), which were introduced into the yeast system, revealed generally higher preference towards tested acyl donors when 18:1-LPE was used as an acyl acceptor compared to assays with 18:2-LPE, at least in 30 °C. With 18:1-LPE, *Cs*LPEAT1a was most specific towards saturated and monounsaturated acyl donors. The gene, which encoded this isoform was predominantly expressed in seeds, while the seed expression of *Cs*LPEAT2 was significantly lower than in other tissues. Due to the governing presence of *Cs*LPEAT1a in seeds its activity may be correlated with preference towards 18:1-LPE in this tissue. This molecular species of LPE is the second most abundant LPE in *C. sativa* seeds (after 16:0-LPE), between 18-24 DAF [48]. This period of seed development is characterized by the highest intensity of lipid accumulation [26,28]. Thus, it could be speculated that synthesized, via LPEAT action, PE is utilized by PDAT to transfer fatty acids from *sn-2* position to DAG producing TAG (which is stored) and 18:1-LPE (which can be further reacylated via LPEAT action). However, high preferences of *Cs*LPEAT1a towards saturated and monounsaturated fatty acids do not fit well with this hypothesis. The fatty acids incorporated into the seeds’ PE pool may be also transferred into PC pool. The PC can be formed from PE through three successive methylations by PE N-methyltransferases (PEMTs). Thus, PEMTs could be additional suppliers of monounsaturated fatty acids to PC pool. Subsequently PC-attached acyl groups can be desaturated. Unsaturated fatty acids are the dominant acyl compounds in *C. sativa* seeds [28,48,55,57]. However, so far not all PEMT enzymes needed for PE conversion to PC were identified in plants.

Contrary to both *Cs*LPEAT1a isoform variants, which preferred utilization of 18:1-LPE, each of *Cs*LPEAT2 isoforms favored 18:2-LPE, as an acyl acceptor. Each isoform was able to incorporate all tested acyl donors into 18:2-LPE. *Cs*LPEAT2c, followed by *Cs*LPEAT2b, displayed the highest activity. Genes encoding these isoforms were the highest and the lowest expressed in vegetative tissues, respectively. Second-highest expressed gene was *Cs*LPEAT1a, which preferred 18:1-LPE. The existence of enzymes with such various properties may be essentially connected with their expression in different plant tissues. Vegetative tissues are much more susceptible to external biotic conditions, which is why greater variability of acyl acceptors and donors may be needed to adapt to altered temperature conditions. Seeds possess additional protection of seed coats and siliques and are mainly responsible for lipid accumulation.

*Cs*LPEAT susceptibility to temperature, which we noticed in enzyme assays conducted in vitro with plant-derived microsomal fractions, was also confirmed for each individual cloned isoform introduced into the yeast system. In the yeast microsomal fraction assays, the 18:1-LPE were used as an acyl acceptor, because both *Cs*LPEAT1 and *Cs*LPEAT2 isoforms efficiently select this LPE type. Towards 16:0-CoA and 18:0-CoA the most active was *Cs*LPEAT2c, regardless of temperature. The largest variability was observed when unsaturated fatty acids were used as acyl donors. At the lowest tested temperature 18:1-CoA and 18:2-CoA were mainly utilized by *Cs*LPEAT2a. This preference, however, changed with temperature increase. At 20 °C, *Cs*LPEAT2c was the most active, whereas with further temperature rise, *Cs*LPEAT2b started to be the more active isoform. In lower temperatures 18:3-CoA was utilized by each *Cs*LPEAT2 isoform with similar efficiency. The temperature rise favored *Cs*LPEAT2b. In addition, other differences between isoforms and preferred acyl donors were detected. Observed *Cs*LPEAT isoforms’ behavior in correspondence with temperature fluctuations, supports our findings that these enzymes play a significant role in adaptation to cold and heat stress, through the adjustment of PE fatty acids composition and consequently membrane fluidity. Moreover, higher or lower expression of genes, encoding individual isoforms, may also correlate with temperature. Further studies are needed to verify this suggestion. Up until the presented study, no substrate specificity, selectivity, and activity assays at different temperatures were conducted for LPLAT enzymes. This work marks the first time that such an in-depth thermal and biochemical characterization of LPEAT enzymes was carried out.

## 4. Materials and Methods

### 4.1. Chemicals

Non-labelled acyl-CoA and radiolabelled [^14^C]acyl-CoA were synthesized from unlabelled fatty acids (Sigma-Aldrich, St. Louis, MO, USA) and ^14^C-labelled fatty acids (Perkin-Elmer) according to a modified method described by Sánchez et al. [60]. Other substrates for enzymes assays: non-labelled *sn-1*-18:1-lysophosphatidylethanolamine, *sn-1*-18:1-lysophosphatidylcholine and lipid standards for thin-layer chromatography were purchased from Larodan Fine Chemicals and Avanti Polar Lipids, respectively.

### 4.2. Plant Material and Growth Condition

The analyzed plant material was derived from *Camelina sativa* L. Crantz, cv. Suneson. For obtaining plant material grown in in vivo, false flax seeds were planted in soil and cultivated in growth chamber at 23 °C with a photoperiod of 16 h of light (120 mmol photons^m−2 s−1^)/8 h of dark with relative humidity of 60%. The day when plants possessed well-developed flower buds, leaf materials were collected and after 24 days parts of their siliques were picked for further seed analysis.

To acquire material from the roots and leaves grown in vitro, *C. sativa* seeds were surface sterilized by immersion in 70% ethanol for 1 min followed by 10 min incubation in 3% calcium hypochlorite. After that, the seeds were washed at least 3 times in distilled water and then planted on plates containing: 0.8% agar, 0.5 × Murashige and Skoog (MS) medium and 2% sucrose. After 10 days, the seedlings were transferred into flasks with liquid 0.5 × MS medium containing 2% sucrose and grown with shaking (100 rpm) for two more weeks at 23 °C.

### 4.3. Gene Cloning and Sequence Analysis

Putative LPEAT sequences were amplified using cDNA derived from developing tissues of *C. sativa*. Due to high similarity of sequences, to find every variant of LPEAT1 and LPEAT2, one pair of gene-specific primers, corresponding to each isoform, were used (Supplementary Table S2).

Phylogenetic tree construction of *Cs*LPEAT isoforms and their variants were performed in MEGA X [61] with 1.000 bootstraps [62] in relation to *Arabidopsis lyrata, Arabidopsis thaliana, Brassica napus, Brassica oleracea, Brassica rapa, Capsella rubella, Eutrema salsugineum,* and *Raphanus sativus*. Their amino acid sequences were obtained from the NCBI databases. Sequence annotation was performed using CLC Main Workbench (Qiagen, Hilden, Germany).

### 4.4. Vector Construction and Yeast Transformation

*Cs*LPEAT1 and *Cs*LPEAT2 isoenzymes were cloned by Gateway cloning according to the manufacturer’s protocol (Thermo Fisher, Waltham, MA, USA). The sequences were first incorporated into pDONR221 plasmids and then into pDEST52 plasmids containing a galactokinase 1 (GAL1) promoter.

Obtained pDEST52-GAL1::*Cs*LPEAT constructs and an empty plasmid control were used for the transformation of *Saccharomyces cerevisiae* haploid knock-out mutants ALE1 (BY4741; Matα, his3Δ1; leu2Δ0; lys2Δ0; ura3Δ0; YOR175c::kanMX4). The transformations of yeast were done according to the modified LiAc/SS carrier DNA/PEG method [63].

### 4.5. Cleavage of Phosphatidylethanolamine by Phospholipase A_2_

Separation of phosphatidylethanolamine class was done according to the described by Klińska et al. [26], for which lipid extracts of 6-weeks old leaves from *C. sativa* were used. Individual lipid classes were visualized, on developed in polar solvent TLC plate, by a brief exposure to iodine vapours. Areas of the gel containing the PE, identified by means of authentic standards, were scraped off after the iodine has completely evaporated. Into the scraped gel fragments mixture of chloroform:methanol (1:2; v:v) was added and sonicated for 15 min. Extracted from gel PE fractions were collected to new probes and equal volumes of acetic acid, chloroform and water were added and centrifuged. The bottom, chloroform fraction containing PE was collected, dried under a stream of N_2_ and dissolved in 0.5 mL of diethyl ether. Subsequently, 1 mL of 0.1 M Tris-HCl (pH 8.9) with 5mM CaCl_2_ and 3.3 U of phospholipase A_2_ (derived from honeybee venom; Sigma-Aldrich, St. Louis, MO, USA) was added and vigorously stirred for 1 h, to increase the surface contact between the water fraction in which phospholipase is present with the diethyl ether fraction containing PE. Reactions were stopped by supplementation of 20 µL of acetic acid. To isolate only lipid fraction containing fatty acids and lysophospatidylethanolamine derived after PLA_2_ activity, double Blight and Dyer (1959) [64] extraction method was applied. 3.75 mL of chloroform:methanol (1:2,*v/v*) followed by the addition of 1.25 mL of 0.15 M acetic acid, 1.25 mL of chloroform and 1.25 mL of water were added to the samples. Collected, after centrifugation, chloroform fractions were separated by TLC on Silica gel 60 plates (Merck, Darmstadt, Germany) in chloroform:methanol:acetic acid:water (90:15:10:2.5, *v/v/v/v*). Separated lipids: free fatty acids and lysophospatidylethanolamine, were visualized on the plate by water spraying. Areas of gel containing the LPE and free fatty acids were scraped off and methylated in situ on the gel with 2% H_2_SO_4_ in dry methanol (60 min at 90 °C). The fatty acid methyl esters were extracted with hexane and the internal standard—methyl-heptadecanoate was added. Analysis of content and fatty acid composition of prepared samples was conducted by gas-liquid chromatography (Shimadzu; GC-2010) equipped with a fame ionization detector (FID) and a 60 m × 0.25 mm CP-WAX 58-CB fused-silica column (Agilent Technologies, Santa Clara, CA, USA).

### 4.6. Plant Microsomal Fraction Isolation and Enzyme Assay

Microsomal membranes were prepared from freshly harvested tissues and isolations were done according to the method previously described [28] and stored at −80 °C for further analysis. Determination of the microsomal fraction concentration was done by phosphatidylcholine (PC) and in some assays phosphatidylethanolamine (PE) content estimations. From the aliquots of derived microsomal fractions, lipid fractions were extracted by Blight and Dyer methods, described above. The chloroform fractions, containing lipids, were collected and separated for individual polar lipid classes by TLC in polar solvent. The plate with separated lipid classes was sprayed by 0.05% primuline solution and visualized under UV light. Based on used lipid standards, areas of gel containing the PC and PE were scraped off and methylated in situ on the gel with 2% H2SO4 in dry methanol (60 min at 90 °C). Derived fatty acid methyl esters were analyzed by gas chromatography as described in the previous paragraph.

Aliquots of microsomal isolates from in vivo leaves were subsequently used in in vitro optimization assays, to determine the best parameters for enzymatic reaction conducted by LPEAT-type enzymes present in vegetative tissue. The effect of various factors, such as reaction time, amount of microsomal fraction and buffer pH were examined in assays were 5 nmol of [^14^C]18:1-CoA and 5 nmol of *sn-1*–18:1-lysophosphatidylethanolamine were added. In case of seeds, the optimal parameters established by Klińska et. al. (2020) have been used.

Study focusing on activity and substrate specificity of LPEAT-type enzymes were performed with five [^14^C]acyl-CoAs: palmitoyl-CoA (16:0), stearoyl-CoA (18:0), oleoyl-CoA (18:1), linoleoyl-CoA (18:2) and linolenoyl-CoA (18:3). To each reactions 5 nmol of *sn-1*–18:1-lysophosphatidylethanolamine (or *sn-1*–16:0-lysophosphatidylethanolamine, or *sn-1*–18:2-lysophosphatidylethanolamine, depending on conducted assays) and 5 nmol of appropriate [^14^C]acyl-CoA were added. Final reaction mixture consisted of (in total volume of 100ul) 0.1M phosphate buffer (pH 7.2), 0.5 nmol of microsomal PC equivalent to 2.2 µg of microsomal proteins and in case of selectivity assay 2 mg/mL of BSA. Reactions were carried out for 60 min at 30 °C (or in 10, 20, 40 °C in assay determining substrate specificity at different temperature) in Eppendorf Thermomixer Compact with continuous shaking (1250 rpm). Termination of the reactions were done by adding 375 µL chloroform/methanol (1:2; *v/v*), 5 µL of glacial acetic acid, 125 µL of chloroform and 125 ul of water. Obtained chloroform fractions, containing lipids, were collected and separated on silica gel 60 plate by thin layer chromatography in polar solvent (chloroform/methanol/acetic acid/water; 90/15/10/2.5; *v/v/v/v*). The separated radiolabelled product of reaction was visualized and quantified by electronic autoradiography (Instant Imager, Packard Instrument Co., Meriden, CT, USA).

LPCAT activity assays were carried out in a similar manner. The only differences were: replacement of *sn-1*-18:1-LPE by *sn-1*–18:1-lysophosphatidylcholine, addition of aliquots of microsomal fraction containing 0.2 nmol microsomal PC and 30 min of reaction time (optimal parameters established by Klińska et al. [28]).

The activity and acyl donor preference of LPEAT enzymes in the backward reaction assays were conducted with addition of 10 nmol of [^14^C]acyl-CoA, 0.2 µmol of free coenzyme A (CoA) and 1 mg of BSA in a total volume of 100 µL of 0.04 M potassium bufer (pH 7.2) with or without 0.5 µmol of dithionitrobenzoic acid (DTNB). Reactions were conducted for 60 min at 30 °C, with addition of 5 nmol of microsomal fractions. Calculation of backward reaction activity and estimation of the time of complete fatty acids turnover in PE pool were conducted according to (Jasieniecka-Gazarkiewicz et al. 2016; [20,28].

### 4.7. Yeast Microsomal Preparation and Assay of CsLPEAT Isoforms Variant

The transformed yeast cells carrying empty plasmid or plasmid with one of the tested LPEAT isoform genes were first cultured in uracil drop-out medium containing 2% of raffinose for 48h at 30 °C on rotating platform (230rpm). Subsequently, these yeast cultures were used to inoculate 100mL of fresh uracil drop-out medium with 2% of raffinose to an absorbance of OD_600_ of 0.2 and were cultivated for next 24h. After that time, the medium was supplemented by galactose to obtain a 2% final concentration and followed by 24h incubation to induce gene expression and protein synthesis. Microsomal fractions were harvested as described Jasieniecka-Gazarkiewicz et.al. [20]. The aliquots of isolated microsomal fractions were examined to determine the phosphatidylcholine (PC) content for further enzymes essay.

Before determining the substrate specificity, the optimization was done for each of LPEAT isoform variants establishing the best time of reaction and amount of microsomal fractions. Further studies concentrated at substrate specificity towards various [^14^C]acyl-CoAs and the determination of activity of each of isoform variants at different temperatures, towards five chosen [^14^C]acyl-CoAs: palmitoyl-CoA, stearoyl-CoA, oleoyl-CoA, linoleoyl-CoA, and linolenoyl-CoA. Assays were conducted in a similar fashion as described above, except of reaction parameters, which were adapted from results of optimization analysis. Additional analyses determining the activity of *Cs*LPEAT against *sn-1*–16:0-lysophosphatidylethanolamine and *sn-1*–18:2-lysophosphatidylethanolamine were done.

### 4.8. Expression Analysis

Primers for RT-qPCR can be found in Supplementary Table S3, along with their reference sequences. For this analysis, plants were cultivated as described in subsection ‘Plant Material and Growth Condition’. Total RNA was extracted from all the tissues with GeneMatrix Universal RNA Purification Kit (EurX). Possible gDNA contamination was removed through incubation with dsDNase (Thermo Fisher Scientific). Maxima First Strand cDNA Synthesis Kit for RT-qPCR (Thermo Fisher Scientific, Waltham, MA, USA) was used to synthesize cDNA from RNA template. Desired cDNA fragments were amplified using Maxima SYBR Green/ROX qPCR Master Mix (2×; Thermo Fisher Scientific) in QuantStudioTM 3 Real-Time PCR System (Applied Biosystems, Waltham, MA, USA). For absolute expression analysis, amplicons of the investigated genes were purified with GeneJET PCR Purification Kit (Thermo Fisher Scientific). Serial dilutions of the amplicons were analyzed with qPCR to create amplification curves, which were later compared to particular genes’ cDNA amplifications. Relative expression was conducted by applying the 2–ΔΔCT algorithm [65]. *Cs*LPEAT1a expression was treated as control, and all the results were normalized to either *Cs*ACT2 or *Cs*TIP41 housekeeping genes. *Cs*LPEAT amplicons were sequenced to confirm the primers’ amplified desired genes.

### 4.9. Sequence Comparison and Evolutionary Analysis

References for amino acid sequences (others than the once isolated and sequenced by us) used in sequence alignments and evolutionary analyses can be found in Supplementary Table S4. The alignments presented in this publication were created in CLC Main Workbench 20 (Qiagen, Hilden, Germany). Domains and substrate-binding sites were found with NCBI Conserved Domain Search. The alignments for evolutionary analyses were created using ClustalW. The evolutionary histories were inferred by using the Maximum Likelihood method and JTT matrix-based model [66]. The bootstrap consensus trees inferred from 1000 replicates each [62] are taken to represent the evolutionary history of the taxa analyzed [62]. Branches corresponding to partitions reproduced in less than 50% bootstrap replicates are collapsed. The percentage of replicate trees in which the associated taxa clustered together in the bootstrap test (1000 replicates) are shown next to the branches [62]. Initial trees for the heuristic search were obtained automatically by applying Neighbor-Join and BioNJ algorithms to a matrix of pairwise distances estimated using the JTT model, and then selecting the topology with superior log likelihood value. LPEAT1 analysis involved 12 amino acid sequences and LPEAT2 analysis involved 11. There was a total of 466 positions in the final dataset for LPEAT1 analysis and 549 for LPEAT2 analysis. Evolutionary analyses were conducted in MEGA X [61].

## 5. Conclusions

Our study revealed that LPEAT enzymes substrate specificity can be modified by temperature. Moreover, we showed that each individual isoform of LPEAT enzymes has unique substrate preferences and the genes encoding these isoforms are showing different expression patterns in different tissues. All of this indicates that the role of LPEAT in plant metabolism and especially in phospholipid remodeling is very complex and can be regulated in many ways (including by environmental conditions). Existence of three isoforms of *Cs*LPEAT1 and three isoforms of *Cs*LPEAT2 has been shown. Sequence cloning allowed for detailed bioinformati*Cs* analyses including the determination of phylogenetic distance between each *Cs*LPEAT isoform and revealed that *Cs*LPEAT1c and *Cs*LPEAT2c are more evolutionary-divergent from two other more-closely related homeologs. This finding suggests that these isoforms originated from *C. hispida*, whereas other isoforms originated from *C. neglecta*.

## Figures and Tables

**Table 1:** Incorporation rate of [^14^C]acyl groups from [^14^C]acyl-CoA into PE of microsomal fractions of *Camelina sativa* in vivo leaves and estimated time of complete exchange of acyl groups of PE for fatty acids from acyl-CoA pool. Mean values and SD are presented (data from at least three independent assays).

Temperature	Acyl-CoA Used in the Assay	pmol [ ^14^C]PE/nmol Microsomal PE/min]	Time of Exchange of All PE Fatty Acids (FA) for FA from Acyl-CoA Pool [Days]
10 °C	[^14^C]18:1-CoA	0.194 ± 0.03	7.2
	[^14^C]18:2-CoA	0.155 ± 0.004	8.9
	[^14^C]18:3-CoA	0.101 ± 0.005	13.8
40 °C	[^14^C]18:1-CoA	0.711 ± 0.03	2.0
	[^14^C]18:2-CoA	0.763 ± 0.007	1.8
	[^14^C]18:3-CoA	0.295 ± 0.02	5.1

## Data Availability

The data presented in this study are available on request from the corresponding authors. The data are not publicly available due to privacy.

## Supplementary Materials

The following are available online at https://www.mdpi.com/article/10.3390/ijms22158137/s1.
