# Supplementary material for: LPEATs Tailor Plant Phospholipid Composition through Adjusting Substrate Preferences to Temperature
Source: Int J Mol Sci. 2021 Jul 29;22(15):8137. doi: 10.3390/ijms22158137 (PMC8348727; doi:10.3390/ijms22158137)
Supplement: Supplementary file 1 [file ijms-22-08137-s001.zip › ijms-1300451-supplementary.pdf]

Supplementals

Supplemental Table S1. Percentage of amino acid similarity between *Cs*LPEAT1a isoform variants (A) and *Cs*LPEAT2 isoforms (B).

**A**	*Cs*LPEAT1a.1	*Cs*LPEAT1a.2
*Cs*LPEAT1a.1		99.24%
*Cs*LPEAT1a.2	99.24%	

**B**	*Cs*LPEAT2a	*Cs*LPEAT2b	*Cs*LPEAT2c
*Cs*LPEAT2a		98.17%	98.17%
*Cs*LPEAT2b	98.17%		97.61%
*Cs*LPEAT2c	97.61%	97.61%	

Supplemental Table S2. Primers used to amplify *Cs*LPEAT genes, later cloned into the Gateway system. attB1 fragments of forward primers and attB2 fragments of reverse primers are underlined

**Gene-of-Interest**	**Primer Role**	**Primer Sequence (5' to 3')**
*Cs*LPEAT1	forward	GGGGACAAGTTTGTACAAAAAAGCAGGCTGTGTTTACCTTTTACGGCGAATCTA
	reverse	GGGGACCACTTTGTACAAGAAAGCTGGGTTCGTGTAATCAAACATTTGGCTTTT
*Cs*LPEAT2	forward	GGGGACAAGTTTGTACAAAAAAGCAGGCTACATTCGACCACAAAGCAAAAAGGC
	reverse	GGGGACCACTTTGTACAAGAAAGCTGGGTAAAGTTTGCTCAGAACTAGAGG

Supplemental Table S3. Primers used in RT-qPCR analysis.

**Gene-of-Interest**	**Reference Sequence**	**Primer Role**	**Primer Sequence (5' to 3')**
*Cs*ACT2	XM_010489499.1	forward	ACAATTTCCCGCTCTGCTGTTGTG
*Cs*ACT2	XM_010489499.1	reverse	AGGGTTTCTCTCTTCCACATGCCA
*Cs*TIP41	XM_019233743.1	forward	TTGGCGAGAATGCATTAGTCCTT
*Cs*TIP41	XM_019233743.1	reverse	ATTGTCGGCCAGTTCATCTTCATA
*Cs*LPEAT1a	XM_010431575.2	forward	GACGACGACGACGAGAAA

*Cs*LPEAT1a	XM_010431575.2	reverse	TAAATGAAAGAAAGGGCTTCAG
*Cs*LPEAT1b	XM_010419341.2	forward	AAAGCTGAAATCTTCACGG
*Cs*LPEAT1b	XM_010419341.2	reverse	CAAAGAAAGAAAGGGGCTT
*Cs*LPEAT1c	XM_010474544.2	forward	CTAGAGACGACGAGGAGGAG
*Cs*LPEAT1c	XM_010474544.2	reverse	CTCTGATTCCATTGTTCTTCTTCTA
*Cs*LPEAT2a	XM_010519887.1	forward	AAGCAAAAAGGCTGTGTCAATC
*Cs*LPEAT2a	XM_010519887.1	reverse	TTCTGGTTGATCGACGGATGG
*Cs*LPEAT2b	XM_010509827.2	forward	TGATCCATCATCAACCAGAG
*Cs*LPEAT2b	XM_010509827.2	reverse	AAGAACAGGAGGCTCTGT
*Cs*LPEAT2c	XM_010508235.2	forward	AGCAAAAAGGCTGTGAGTAC
*Cs*LPEAT2c	XM_010508235.2	reverse	TGGATGATTGATGATGGATCAAC

Supplemental Table S4. List of reference sequences used for sequence alignments and evolutionary analyses.

Gene Denotation	Reference Sequence	Species of Origin
*Cs*LPEAT1b	XM_010419341.2	*Camelina sativa*
*Cs*LPEAT1c	XM_010474544.2	*Camelina sativa*
*At*LPEAT1	AT1G80950.1	*Arabidopsis thaliana*
*Al*LPEAT1	XP_020889625.1	*Arabidopsis lyrata*
*Bn*LPEAT1.1	XP_013702411.1	*Brassica napus*
*Bn*LPEAT1.2	XP_013648598.1	*Brassica napus*
*Bo*LPEAT1	XP_013591940.1	*Brassica oleracea*
*Br*LPEAT1	XP_009104503.2	*Brassica rapa*
*Es*LPEAT1	XP_006389853.1	*Eutrema salsugineum*
*Rs*LPEAT1	XP_018457802.1	*Raphanus sativus*
*Cs*LPEAT2a	XM_010519887.1	*Camelina sativa*
*Cs*LPEAT2b	XM_010509827.2	*Camelina sativa*
*Cs*LPEAT2c	XM_010508235.2	*Camelina sativa*
*At*LPEAT2	AT2G45670.1	*Arabidopsis thaliana*
*Al*LPEAT2	XP_002882025.1	*Arabidopsis lyrata*
*Bn*LPEAT2.1	XP_013734815.1	*Brassica napus*
*Bn*LPEAT2.2	XP_022571765.1	*Brassica napus*
*Bo*LPEAT2	XP_013631253.1	*Brassica oleracea*
*Br*LPEAT2	XP_009133738.1	*Brassica rapa*
*Es*LPEAT2	XP_006397746.1	*Eutrema salsugineum*
*Rs*LPEAT2	XP_018484721.1	*Raphanus sativus*

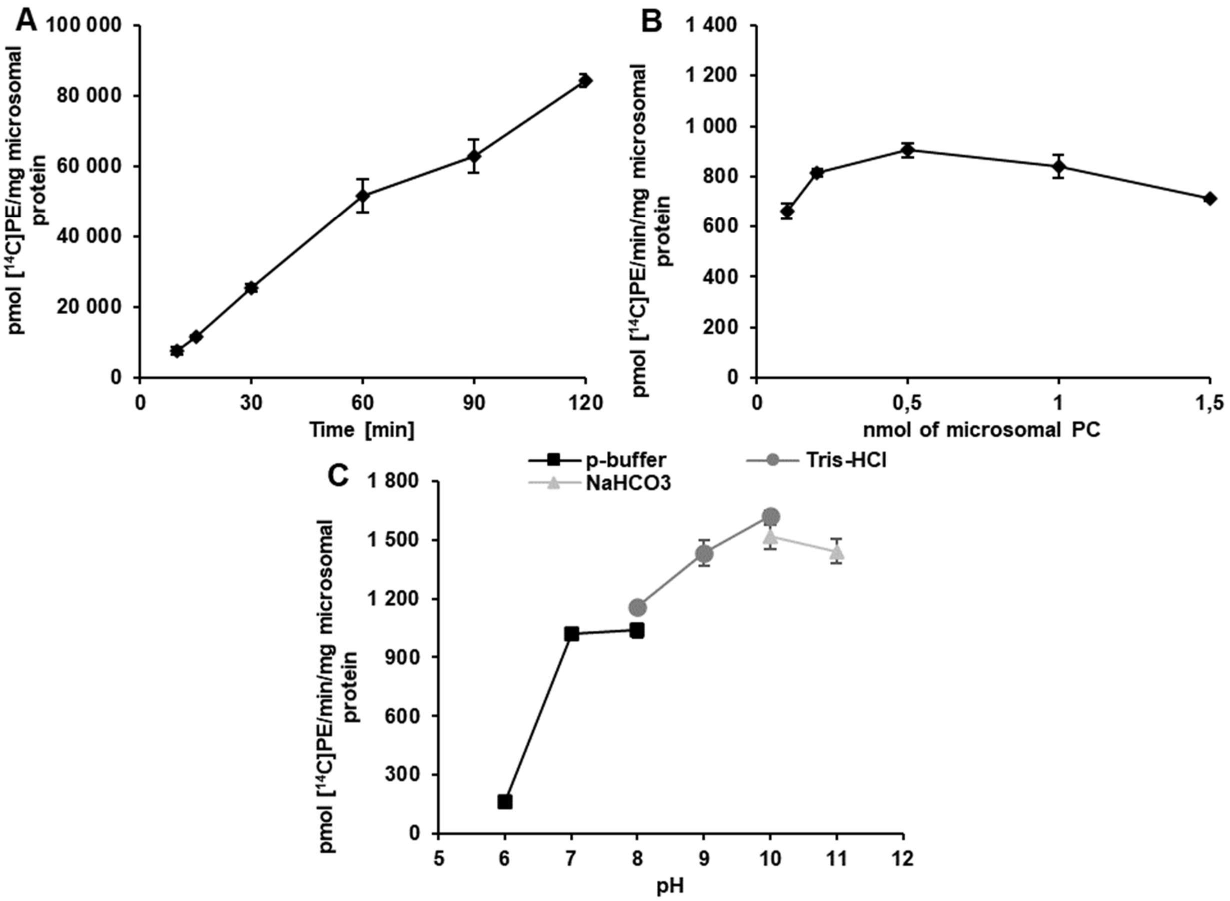

Supplemental Figure S1. Effects of various factors on the activity of acyl-CoA:lysophosphatidylethanolamine acyltransferases (LPEATs) of *Camelina sativa in vivo* leaves. A – time dependency; B – microsomal content dependency; C - pH dependency. Mean values and SD are presented (data from at least three independent assays).

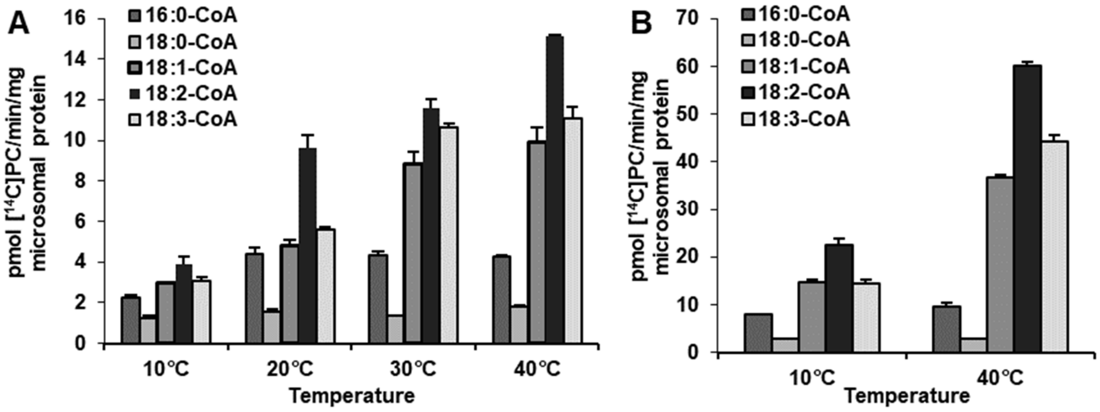

Supplemental Figure S2. Activity of acyl-CoA:lysophospatidylcholine acyltransferases (LPCATs) present in microsomal fraction of *Camelina sativa* tissues towards five various acyl-CoAs at different temperatures. (A – *in vivo* leaves; B – *in vitro* roots). Mean values and SD are presented (data from at least three independent assays).

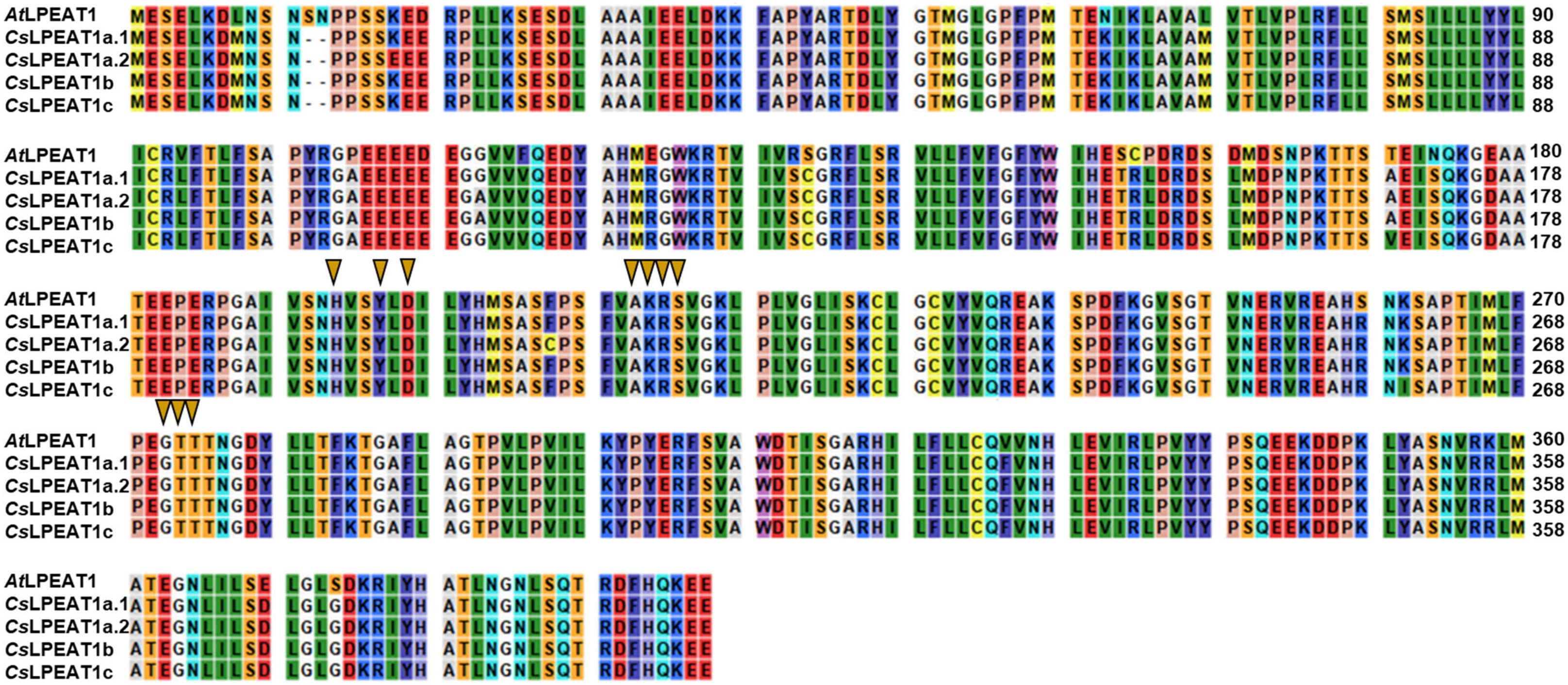

Supplementary Figure S3. Amino acid sequences' annotation of *Cs*LPEAT1 isoforms and *At*LPEAT1. LPE-binding site amino acids are denoted by golden arrows above.

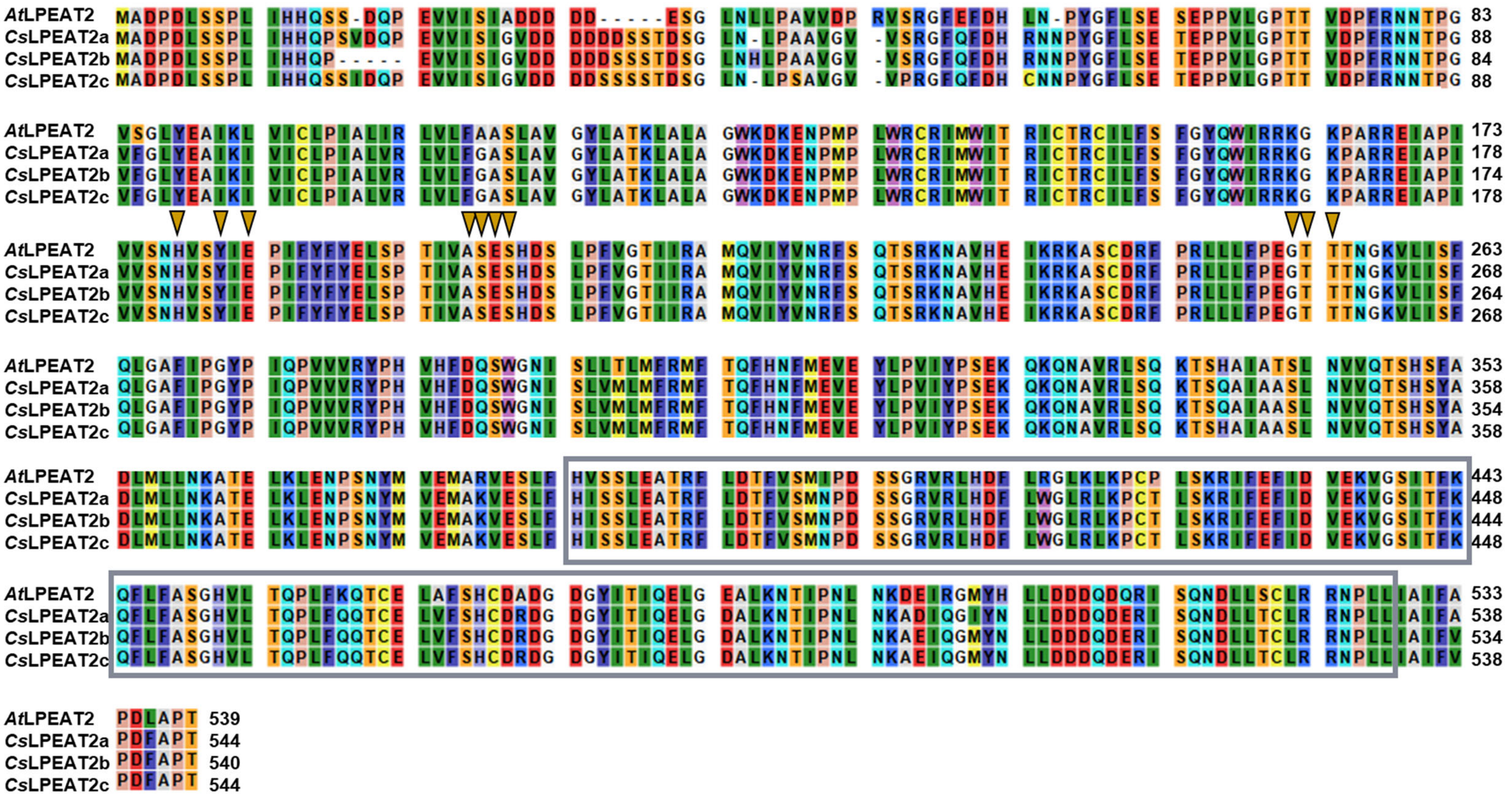

Supplementary Figure S4. Amino acid sequences' annotation of *Cs*LPEAT2 isoforms and *At*LPEAT2. LPE-binding site amino acids are denoted by golden arrows above. Ca^{2+}-binding protein domain is framed by a silver line.

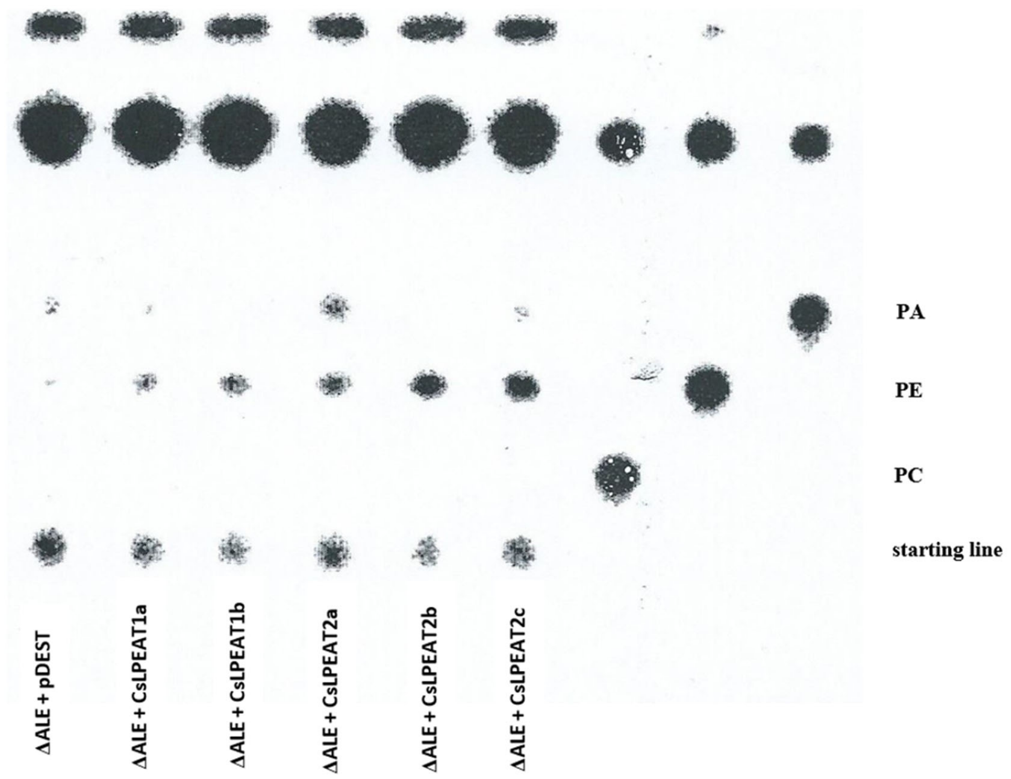

Supplemental Figure S5. Complementation of PE synthesis *via* LPLAT pathway in *S. cerevisiae* transformants overexpressed with cloned *Cs*LPEAT1a.1, *Cs*LPEAT1a.2, *Cs*LPEAT2a, *Cs*LPEAT2b and *Cs*LPEAT2c isoforms. Figure presents the results of radioisotope assay analysed by Instant Imager, Packard Instrument Co. Assay was done with addition of 18:1-LPE and 18:1-CoA. The first line presents negative control (*ΔALE* strain) incapable to synthesize PE *via* LPLAT pathway. Radiolabeled standards of each phospholipids were obtained via enzymatic reaction conducted with wild strain BY4742.

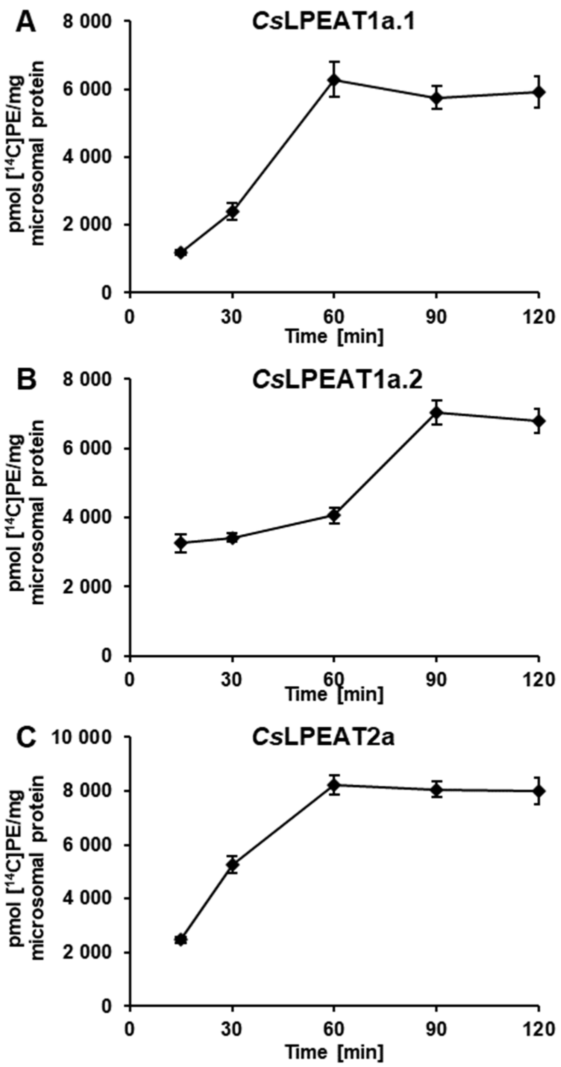
A
CsLPEAT1a.1
pmol [14C]PE/mg microsomal protein
8 000
6 000
4 000
2 000
0
0
30
60
90
120
Time [min]
B
CsLPEAT1a.2
pmol [14C]PE/mg microsomal protein
8 000
6 000
4 000
2 000
0
0
30
60
90
120
Time [min]
C
CsLPEAT2a
pmol [14C]PE/mg microsomal protein
10 000
8 000
6 000
4 000
2 000
0
0
30
60
90
120
Time [min]

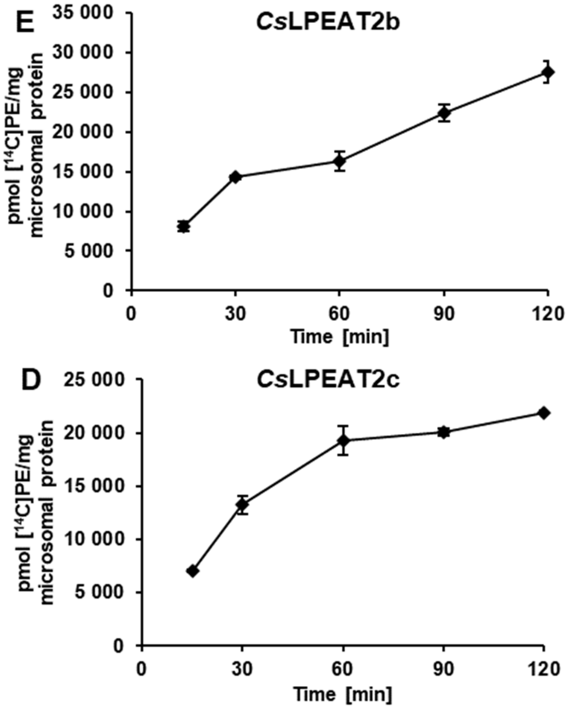

Supplemental Figure S6. Effects of reaction time on the activity of various variants of acyl-CoA:lysophosphatidylethanolamine acyltransferases (LPEATs). Mean values and SD are presented (data from at least three independent assays).

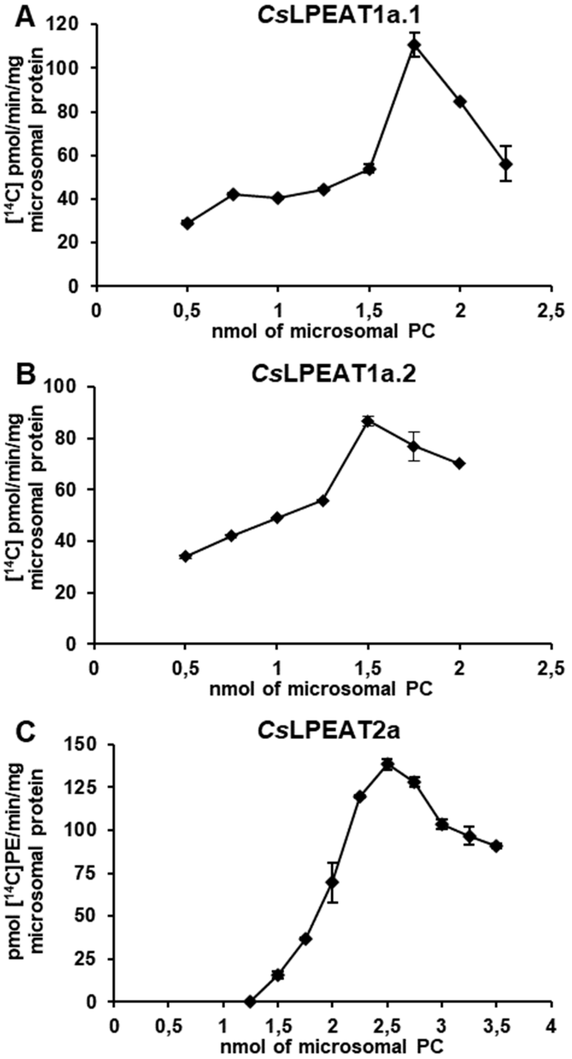
A
CsLPEAT1a.1
[¹⁴C] pmol/min/mg microsomal protein
nmol of microsomal PC
B
CsLPEAT1a.2
[¹⁴C] pmol/min/mg microsomal protein
nmol of microsomal PC
C
CsLPEAT2a
pmol [¹⁴C]PE/min/mg microsomal protein
nmol of microsomal PC

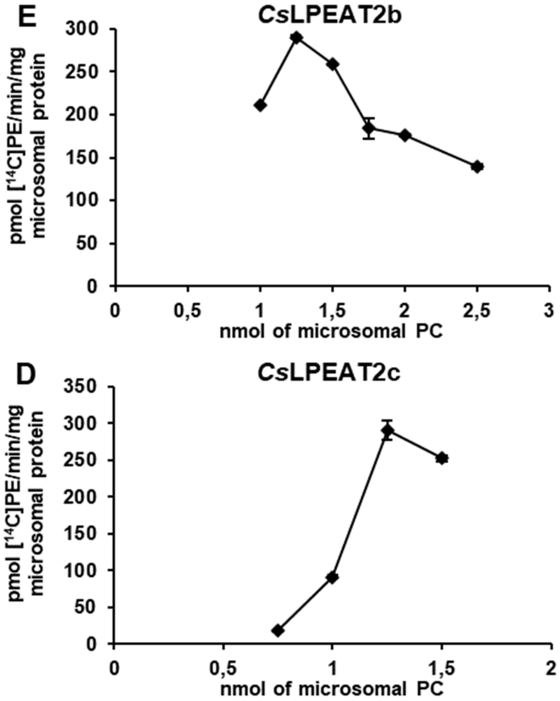

Supplemental Figure S7. Effects of amount of microsomal fraction on the activity of various isoforms of acyl-CoA:lysophosphatidylethanolamine acyltransferases (LPEATs). Mean values and SD are presented (data from at least three independent assays).
